# Myocardial fibrosis and tissue alterations predict cardiovascular outcomes in chronic kidney disease—a prospective virtual twin study design using large-scale population database

**DOI:** 10.3389/fcvm.2026.1726445

**Published:** 2026-05-11

**Authors:** Georgios Vavilis, Yeshe M. Kway, Zahra Raisi-Estabragh, Steffen E. Petersen, Stefan Neubauer, Qiang Zhang, Vanessa M. Ferreira, Stefan K. Piechnik

**Affiliations:** 1 Division of Cardiovascular Medicine, Radcliffe Department of Medicine, Oxford Centre for Clinical Magnetic Resonance Research, University of Oxford, Oxford, United Kingdom; 2 Heart and Vascular Theme, Division of Coronary and Valvular Heart Disease, Karolinska University Hospital, Stockholm, Sweden; 3 Barts Heart Centre, St Bartholomew’s Hospital, Barts Health NHS Trust, West Smithfield, London, United Kingdom; 4 NIHR Barts Biomedical Research Centre, William Harvey Research Institute, Queen Mary University of London, Charterhouse Square, London, United Kingdom; 5 Big Data Institute, Li Ka Shing Centre for Health Information and Discovery, University of Oxford, Oxford, United Kingdom

**Keywords:** atrial fibrilatin, cardiac magnet resonance imaging, chronic kidney disease, CKD, heart faiIure, myocardial architecture, myocardial fi- brosis, non-parametric mapping

## Abstract

**Background:**

Myocardial fibrosis is a key feature of chronic kidney disease (CKD) and may contribute to its disproportionate cardiovascular (CV) burden. Cardiovascular magnetic resonance (CMR) T1-mapping quantifies diffuse fibrosis non-invasively, but its prognostic value in CKD remains uncertain

**Aims:**

To investigate associations between native myocardial T1, mortality and incident CV outcomes, in CKD using a virtual twin-matching framework within the UK Biobank imaging cohort.

**Methods:**

We conducted a 1:1 virtual twin-matched case-cohort study of CKD cases and phenotypically matched non-CKD controls based on demographics, comorbidities, socioeconomic status, and cardiovascular risk factors. Paired analyses compared myocardial T1 between groups, and stratified Cox models estimated the effect of a 1-SD T1 increase on incident outcomes over a median follow-up of 4.9 years.

**Results:**

Among 193 matched pairs (median age 68 years; 57.5% women), CKD participants had trend-level higher myocardial T1 values than controls (*p* = 0.063), with greater differences among those experiencing adverse outcomes. Over follow-up, CKD participants had higher cumulative incidences of all-cause mortality (11.3 vs. 3.8 events/1,000 person-years; *p* = 0.049) and heart failure (12.7 vs. 3.9 events/1,000 person-years; *p* = 0.021). In CKD, each 1-SD increase in T1 was associated with higher risks of cardiovascular death (HR: 3.86; 95% CI: 1.62, 9.18), heart failure (HR: 2.00; 95% CI: 1.27, 3.15), and atrial fibrillation (HR: 2.04; 95% CI: 1.01, 4.12), but not all-cause mortality or myocardial infarction. No significant associations were observed in matched non-CKD controls.

**Conclusions:**

Elevated native myocardial T1 was independently associated with CV death, heart failure, and atrial fibrillation in CKD. Virtual twin-matching framework improved comparability and internal validity in matched pairs supporting the precision-matched cohort designs for mechanistic inference and risk stratification in multimorbid populations.

## 1 Introduction

Chronic kidney disease (CKD) is a major global health challenge, affecting over 850 million people worldwide (approximately 10% of the global population) (1). Cardiovascular disease (CVD) is the leading cause of mortality in CKD, with individuals at early disease stages being more likely to die from CVD than to progress to end-stage kidney disease (2). While traditional cardiometabolic risk factors contribute to this burden, they fail to fully explain the markedly elevated cardiovascular risk in CKD, suggesting the presence of disease-specific mechanisms such as diffuse, and chronic inflammation, vascular calcification and myocardial fibrosis (3).

Interstitial myocardial fibrosis is a hallmark of CKD-related remodeling. It progresses to irreversible myocardial scarring and may persist even after kidney transplantation (4). This fibrotic process leads to ventricular stiffening, diastolic dysfunction, heart failure, and sudden cardiac death (3, 5, 6). Cardiovascular magnetic resonance (CMR) with late gadolinium enhancement (LGE) is the gold standard for detecting focal replacement fibrosis. However, its sensitivity for identifying diffuse interstitial fibrosis, including in CKD, is limited (7). Unlike LGE, myocardial T1-mapping can non-invasively and directly quantify diffuse interstitial fibrosis on a pixel-by-pixel basis in high resolution, providing a more sensitive and quantitative marker of myocardial tissue abnormalities (7, 8). Importantly, native (or pre-contrast) T1-mapping does not require administration of gadolinium-based contrast media and is particularly advantageous in the CKD populations by eliminating any risk of gadolinium accumulation in tissues and nephrogenic systemic fibrosis (NSF) (9).

Emerging evidence suggests that myocardial T1 relaxation times are consistently elevated across CKD stages, though prior studies often involved small, highly selected populations (e.g., excluding diabetic CKD or CAD patients), limiting generalizability to the broader CKD spectrum (7, 8, 10). Even though T1 mapping is histologically validated as a fibrosis marker (11) and robustly predicts cardiovascular events and mortality in the general population (12), whether these associations extend to CKD remains uncertain. CKD is characterized by distinct remodeling trajectories, where myocardial fibrosis is more diffuse and widespread (8). Furthermore, competing risks and CKD-specific pathways of sudden cardiac death, arrhythmia, and non-atherosclerotic CVD may further alter the prognostic role of T1 (13).

To address these uncertainties, we leveraged large-scale imaging data from the UK Biobank and implemented a 1:1 virtual twin matching framework aiming to: (1) assess the relationship between CKD and myocardial T1 in individuals with CKD compared with non-CKD matched controls, using data from the UK Biobank cohort, and (2) evaluate whether prolonged myocardial T1 is independently associated with key incident cardiovascular diseases and mortality outcomes in CKD. Each CKD case was paired with a phenotypically matched non-CKD control—balanced across demographics, comorbidities, socioeconomic status, and cardiovascular risk factors—to enhance internal validity and isolate the independent effect of kidney dysfunction on myocardial tissue characteristics and clinical outcomes. This design was specifically selected over conventional 1:3 or 1:4 matching strategies, which can improve statistical power but at the cost of covariate balance, risking residual confounding in complex multimorbid populations such as CKD (14–16). Conceptually, this mirrors the precision-medicine paradigm of digital twins, which aim to improve individualized risk prediction by tailoring comparisons to patient-specific contexts (17). Applying this principle at a population scale represents a methodological shift in cardiovascular epidemiology—providing a framework for CKD-specific risk stratification that integrates myocardial fibrosis biomarkers into prognostic modeling.

## 2 Methods

### 2.1 Study population

This analysis was conducted within the UK Biobank Imaging subcohort, which is derived from the larger UK Biobank prospective, population-based cohort of over 500,000 participants recruited between 2006 and 2010 (18). Participants aged 40–69 years at baseline were invited from across the United Kingdom. In 2014, the UK Biobank Imaging Study was initiated to scan approximately 20% of the cohort (19), using a standardized protocol (20) that includes cardiovascular CMR imaging. The present study uses data collected up to October 31, 2022.

### 2.2 Ethics statement

The study complies with the Declaration of Helsinki. Ethical approval for UK Biobank was covered by the NHS National Research Ethics Service on June 17, 2011 (Ref 11/NW/0382) and extended on June 18, 2021(Ref 21/NW/0157). All participants provided written informed consent (21).

### 2.3 Exposure definition: chronic kidney disease (CKD)

CKD stages 3–5 were defined from linked hospital records using CKD-related ICD-9/10 and the Office of Population Censuses and Surveys Classification of Surgical Operations and Procedures version 4 (OPCS-4) codes recorded before the imaging date. Per KDIGO, CKD definition requires ≥2 abnormal kidney-function measurements ≥90 days apart; Because repeat labs were available for only a small subset of the UK Biobank imaging cohort, we relied on diagnostic codes for the definition of CKD, as single creatinine or eGFR values reflect one time point and cannot establish chronicity (22).

### 2.4 Control definition and study sample construction

The control group was selected from UK Biobank participants with no diagnostic codes indicating CKD stages 3–5 recorded at or before the imaging date.

### 2.5 Inclusion and exclusion criteria

**Figure 1:**
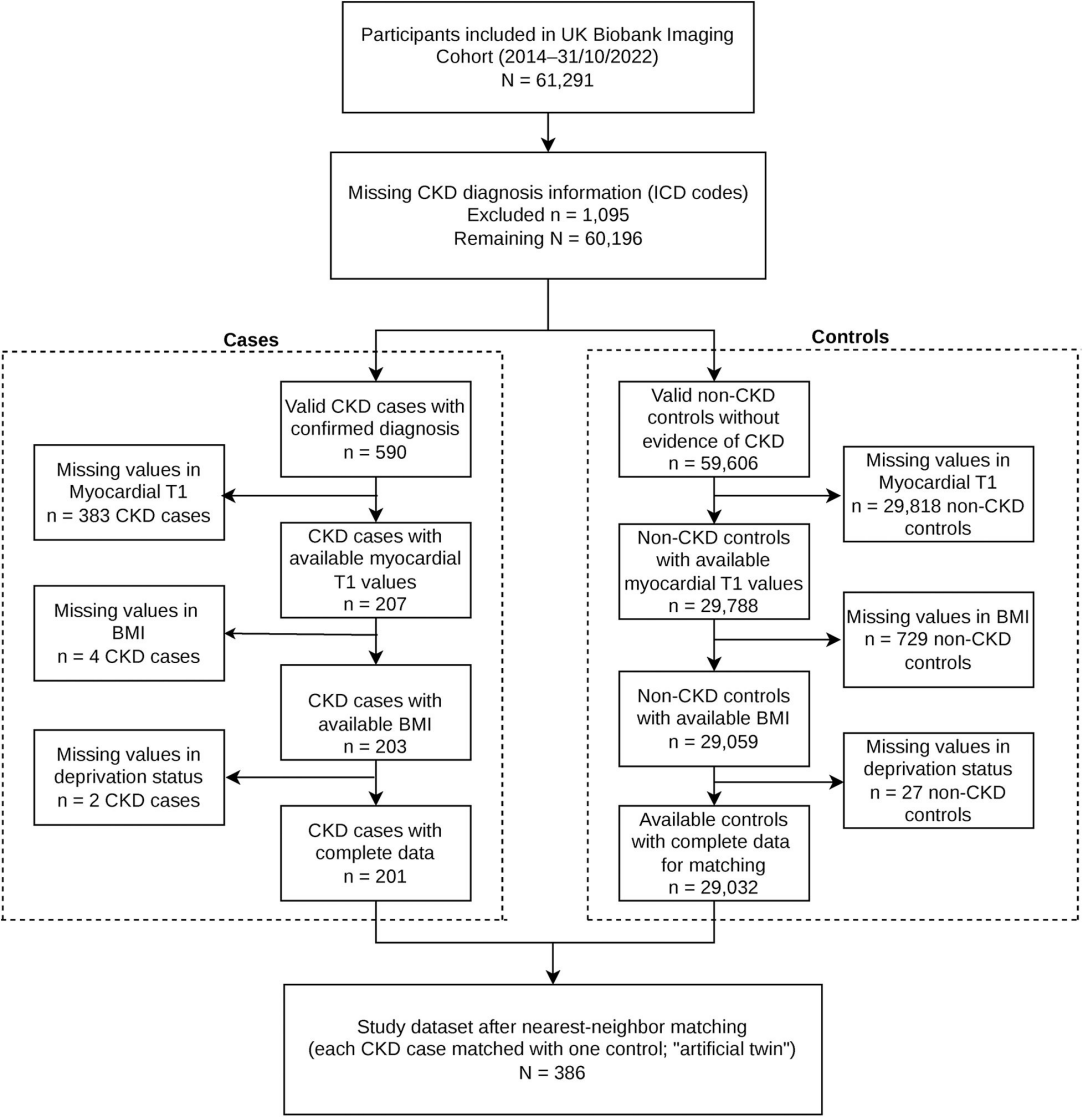
Study recruitment flow chart.

From the full imaging cohort, we excluded participants with missing information in CKD status, missing values for native T1 mapping data, body mass index (BMI), socioeconomic status (Townsend index), or other matching covariates. After applying these criteria (Figure 1), a total of 201 participants met criteria for CKD stages 3–5, and 29,032 participants with preserved kidney function were eligible for inclusion in the control pool. Final analytical samples were defined through matched case-control selection as described in the Matching Methods section.

### 2.6 CMR image acquisition and native myocardial T1 analysis

CMR scans were acquired using standardized 1.5-T scanners (MAGNETOM Aera, Syngo Platform VD13A, Siemens Healthcare) scanners at three dedicated imaging centers in the U.K. (23). Native myocardial T1 mapping was acquired using a single midventricular short-axis slice using the ShMOLLI (Shortened Modified Look-Locker Inversion recovery) sequence (WIP780B) (24). Average myocardial T1 relaxation time was calculated across the slice using a fully automated quality-controlled pipeline; studies with a predicted Dice score of <0.7 were excluded, as previously published (25).

### 2.7 Definition of clinical covariates

All primary clinical covariates arise from the UKBB database at the time of the imaging visit. The following prevalent conditions were included as covariates in this analysis: cardiovascular disease (including valvular heart disease, heart failure, non-ischemic cardiomyopathies, cardiac arrhythmias, atrial fibrillation, myocardial infarction, and ischemic heart disease), stroke, hypertension, diabetes mellitus, hyperlipidemia, and smoking. Comorbidity status was defined using a combination of baseline self-reported diagnoses, International Classification of Diseases 9th and 10th Revision (ICD-10) codes from linked hospital records, OPCS-4 classification systems and clinical data collected during assessment center visits available up to 2014.

Hypertension, diabetes, and hyperlipidemia were defined based on the presence of a recorded diagnosis from any source available up to the imaging visit (self-reported doctor-diagnosed disease, linked hospital inpatient ICD-9/10 codes, and clinical assessment data). Hyperlipidemia was treated as persistent: participants were classified as having hyperlipidemia at baseline regardless of status at the imaging visiting.

#### 2.7.1 Ethnic Q-risk

Ethnicity was self-reported in UK Biobank (26). The ethnic Q-risk algorithms utilized broad ethnic categories to adjust for cardiovascular risk predictions. These categories included: White, South Asian (Indian, Pakistani, Bangladeshi, other Asian background), Black African, Black Caribbean, Chinese, and Other Ethnic Groups. According to UK Biobank documentation, missing or ambiguous data in this variable were handled using imputation techniques during dataset preprocessing (27, 28).

#### 2.7.2 Deprivation townsend index

Area-level socioeconomic status of participants was captured using the 2011 Townsend deprivation index score. This index is an unweighted z-score composite of 4 UK Census variables (rates of unemployment, overcrowding, private vehicle ownership, and home ownership) measured in each census output areas community and compared with national averages per census output areas community in England and Wales (29). A higher value of the Townsend index indicates higher material deprivation, and a lower value indicates relative affluence.

### 2.8 Definition of participant outcomes and follow-up

Primary outcomes were all-cause and cardiovascular mortality, defined via linkage to the national mortality register data (Supplementary Table S1). Participants' health events were tracked prospectively through linkage with national databases, including Hospital Episode Statistics and the Office for National Statistics (12). Secondary outcomes included incident events of atrial fibrillation, heart failure, and myocardial infarction, identified through Hospital Episode Statistics and the Office for National Statistics. The study follow-up started from the date of CMR imaging (index date) and ended at outcome occurrence, loss of follow-up or the study end date.

### 2.9 Study design

An exposure-based matched cohort design was used, emulating a “twin study” approach, to minimize confounding factors and isolate the effect of CKD on myocardial T1 relaxation times and study outcome (28). Each CKD case was matched 1:1 to a non-CKD control using strict multidimensional matching criteria, incorporating demographic, anthropometric, clinical, and socioeconomic factors, leading to a highly curated subset of CKD participants and matched controls. This approach aimed to generate a highly phenotypically comparable subset of participants, enabling more accurate estimation of CKD-specific effects on myocardial tissue characteristics and cardiovascular risk.

#### 2.9.1 Case matching framework

Covariates were selected based on clinical relevance and included both continuous (age, BMI, Townsend Deprivation index) and categorical covariates (sex, ethnicity, hypertension, diabetes mellitus, hyperlipidemia, smoking status, history of cardiovascular disease, and prior stroke) (27). Exact matching was performed for all categorical variables, ensuring each CKD cases was paired with a non-CKD control with identical values for these characteristics (28).

#### 2.9.2 Distance calculation and matching implementation

Continuous variables (age and BMI) were standardized to z-scores. Townsend Deprivation Index was used as provided (already an unweighted z-score composite) (29). Matching was based on Euclidean distance across standardized continuous variables, with a caliper threshold of ±1 standard deviation applied to each variable to ensure close matches (30). Nearest-neighbor matching was conducted in a 1:1 ratio without replacement, ensuring that each CKD case was matched to the most similar non-CKD control available within the defined constraints (27, 31).

#### 2.9.3 Twin control-control matching strategy

Additional “twin-matching” strategy was implemented within the control group. Each identified non-CKD twin was matched to another non-CKD individual with an identical comorbidity and demographic profile using the same matching criteria. This allowed us to assess within-group heterogeneity and distinguish true exposure-related effects from random interindividual variation.

## 3 Statistical analysis

Post-matching balance between CKD and non-CKD participants was assessed using standardized mean differences (SMDs), with values less than 0.10 considered indicative of acceptable covariate balance (32). Paired statistical tests were used to assess whether matched pairs were well balanced and evaluate whether any systematic differences remained after matching: the Wilcoxon signed-rank test for continuous variables and McNemar's test for categorical variables.

To evaluate matching performance, pre- and post-matching *p*-values were compared using Wilcoxon rank-sum tests (continuous variables) and Fisher's exact test (categorical variables). Improvement in balance was quantified using log-transformed *p*-values, and correlations between pre- and post-matching *p*-values were assessed with Spearman's rank and Kendall's tau (33).

### 3.1 Native myocardial T1 value comparisons

Within-pair differences in native myocardial T1 values were evaluated for the entire matched cohort as well as in clinically defined outcome-based subgroups in matched pairs where the CKD participants experienced specific adverse events. Medians, means, IQRs or SDs, and 95% confidence intervals (CI) were reported accordingly.

### 3.2 Association between myocardial T1 and clinical outcomes

Cumulative incidence rates were calculated for each clinical outcome (all-cause mortality, cardiovascular mortality, myocardial infarction, heart failure, and atrial fibrillation) in CKD participants and matched controls.

In the primary analysis, we estimated the association between a 1-standard deviation (SD) increase in myocardial T1 and each outcome separately within the CKD group and the matched non-CKD control group. Hazard ratios (HRs) and 95% confidence intervals (CIs) were calculated for each subgroup to describe group-specific associations. To address potential small-sample bias in endpoints with sparse events (<10 events per outcome), penalized Cox regression using Firth's correction was performed. For cardiovascular mortality, competing-risk analysis was additionally conducted using the Fine–Gray subdistribution hazard model.

In secondary analyses, effect modification by CKD status was formally tested by including an interaction term between T1 and CKD status (T1 × CKD status) in Cox proportional hazards models stratified by matched pairs.

All tests were two-sided, with statistical significance defined as *p* < 0.05 and trend-level significance as *p* < 0.1. Analyses were performed using R version 4.4.2 (R Foundation for Statistical Computing).

## 4 Results

### 4.1 Baseline characteristics of CKD and non-CKD individuals before matching

Before matching, there were significant differences between CKD and non-CKD participants in multiple demographic and clinical variables, including age, BMI, hypertension, hyperlipidemia, and cardiac disease, indicating substantial baseline imbalance between the groups.

#### 4.1.1 Demographics and general characteristic

CKD participants in UKBB were older than those without CKD [median age 67 (IQR: 62, 73) vs. 64 [57, 69] years; *p* < 0.001], more often women (56.7% vs. 48.0%; *p* = 0.017) and more socioeconomic deprivation (median −2.0 vs. −2.7; *p* = 0.012), and a higher median BMI (28 vs. 26 kg/m²; *p* < 0.001). Distribution across ethnic Q-risk categories also differed significantly between groups (*p* < 0.001), with a greater proportion of non-white/non-stated ethnicities in the CKD group (Supplementary Table S3).

#### 4.1.2 Comorbidities and medication use

CKD participants had a significantly higher prevalence of comorbidities, including hypertension (71.1% vs. 32.6%; *p* < 0.001), hyperlipidemia (58.7% vs. 28.3%; *p* < 0.001), diabetes (20.4% vs. 5.5%; *p* < 0.001), prior cardiac disease (33.3% vs. 11.6%; *p* < 0.001), and stroke (7.5% vs. 2.1%; *p* < 0.001). Medication use was substantially higher among CKD participants compared with controls, including beta-blockers (28.4% vs. 6.9%), ACE inhibitors (32.3% vs. 11.2%), ARBs (22.4% vs. 5.8%), diuretics (16.4% vs. 5.3%), calcium channel blockers (30.3% vs. 10.1%), mineralocorticoid receptor antagonists (2.0% vs. 0.3%), nitrates (6.0% vs. 0.8%), lipid-lowering agents (51.2% vs. 22.5%), antithrombotics (36.3% vs. 15.3%), anticoagulants (6.0% vs. 0.9%), and antihyperglycemics (11.4% vs. 3.4%), with *p* < 0.001 for all comparisons (Supplementary Table S3).

#### 4.1.3 Ventricular structure and function

Left ventricular (LV) mass was higher in CKD participants (median 89 vs. 83 g; *p* = 0.042), with greater wall thickness (WT: 5.8 vs. 5.6 mm; *p* = 0.001). Indexed measures showed trends toward higher LV mass and lower stroke volume in CKD, while indexed LV stroke volume (45 vs. 47 mL/m^2^; *p* = 0.016) and cardiac output index (2.7 vs. 2.9 L/min/m^2^; *p* = 0.001) lower in CKD. Most other LV volumetric and ejection fraction measures were comparable between groups (Supplementary Table S3).

Right ventricular (RV) volumes and function were mostly similar, though CKD participants had slightly lower indexed RV stroke volume (45 vs. 47 mL/m^2^; *p* = 0.007) and lower indexed RV end diastolic volume (81 vs. 82 mL/m^2^; *p* = 0.025) (Supplementary Table S3).

#### 4.1.4 Atrial structure and function

The left atrium (LA) was larger in the CKD group compared to the non-CKD group (LA minimal volume: 31 vs. 27 mL; *p* = 0.013), while LA ejection fraction was lower (60% vs. 61%; *p* = 0.002). Indexed LA minimal volume and indexed LA stroke volume also showed modest differences (*p*-values 0.031 to 0.040).

The right atrium (RA) in the CKD group had lower ejection fraction (44% vs. 47%; *p* = 0.002), stroke volume (36 vs. 38 mL; *p* = 0.006), and indexed stroke volume (19 vs. 21 mL/m^2^; *p* < 0.001) compared to the non-CKD group.

### 4.2 Effectiveness of matching and post-matching comparisons

After matching, no significant differences remained in demographic or clinical characteristics (all *p*-values > 0.05), confirming successful balance achieved between CKD and non-CKD participants in these parameters (Table 1). The correlation between pre- and post-matching *p*-values using log-transformed values was weak negative and non-significant (Spearman's rho = −0.21, *p* = 0.52; Kendall's tau = −0.18, *p* = 0.48). Thus, the observed differences before matching did not persist in magnitude or direction after matching, supporting the effectiveness of matching in minimizing systematic differences between groups. Standardized mean differences (SMDs) for all matching covariates fell within acceptable thresholds (<0.1) (32), indicating minimal residual bias and adequate covariate balance (Supplementary Figure S2).

**Table 1:** Baseline characteristics of the study population stratified by the presence of chronic kidney disease (CKD).

Covariates	Non-CKD group (*N* = 193)	CKD group (*N* = 193)	*P*-value	SMD
Sex, %	111 (57.5%)	111 (57.5)	1.000	<0.001
Age	68.0 (62.0, 73.0)	68.0 (62.0, 73.0)	0.961	<0.001
BMI, kg/m^2^	27.5 (25, 31)	28 (25, 31)	0.964	0.06
Smoking, %	4 (2.1)	4 (2.1)	1.00	<0.001
Ethnic Qrisk imp %				
1	184 (95.3)	184 (95.3)	1.00	<0.001
2	1 (0.5)	1 (0.5)	1.00	<0.001
9	8 (4.1)	8 (4.1)	1.00	<0.001
Deprivation Townsend	−2.3 (−3.6, −0.2)	−2.1 (−3.7, 0.1)	0.775	0.1
Diabetes, %	34 (17.6)	34 (17.6)	1.00	<0.001
Hyperlipidemia, %	111 (57.5)	111 (57.5)	1.00	<0.001
Hypertension, %	136 (70.5)	136 (70.5)	1.00	<0.001
Any baseline Cardiac disease, %	62 (32.1)	62 (32.1)	1.00	<0.001
Stroke, %	13 (6.7)	13 (6.7)	1.00	<0.001
Clinical outcomes				
Myocardial Infarction, %	11 (6.7)	5 (3.3)	0.204	0.16
Atrial Fibrillation, %	6 (3.4)	6 (3.4)	1.00	0.01
Heart Failure, %	4 (2.1)	12 (6.4)	0.071	0.21
Cardiovascular Mortality, %	2 (1.00)	3 (1.6)	1.00	0.05
Overall mortality, %	4 (2.1)	11 (5.7)	0.11	0.19
Imaging biomarkers				
Global LV T1, ms	919 (889, 952)	929 (893, 958)	0.097	0.2
LVEDV, mL	146 (129, 169)	146.8 (122, 170)	0.699	0.03
LVEDVi, mL/m^1.7^	78 (69, 87)	77 (67, 86)	0.439	0.05
LVESV, mL	60 (50, 73)	58 (47, 76)	0.522	0.06
LVESVi, mL/m^1.7^	32 (27, 37)	30 (26, 37)	0.327	0.15
LVSV, mL	86 (76, 98)	86 (73, 100)	0.589	0.01
LVEF, %	59 (56, 63)	59 (55, 63)	0.649	0.13
LVCO, L/min	5.4 (4.8, 6.1)	5.2 (4.3, 6.0)	**0** **.** **026**	0.22
LVSVi, mL/m^1.7^	45 (41, 52)	45 (40, 51)	0.351	0.01
LVCOi, L/min/ m^1.7^	2.9 (2.5, 3.2)	2.7 (2.4, 3.1)	**0** **.** **006**	0.29
LVM, g	87 (76, 105)	89 (71, 104)	0.646	0.09
LVMi, g/ m^1.7^	47 (41, 53)	46 (40, 53)	0.379	0.07
WT, mm	5.9 (5.4, 6.5)	5.8 (5.3, 6.5)	0.735	0.08
WTi, mm/ m^1.7^	3.1 (2.9, 3.3)	3.1 (2.8, 3.3)	0.726	0.02
RVEDV, mL	153 (134, 180)	155 (129, 177)	0.332	0.05
RVEDVi, mL/m^1.7^	82 (75, 90)	81 (69, 91)	0.219	0.08
RVESV, mL	69 (56, 80)	65 (53, 79)	0.198	0.18
RVESVi, mL	35 (30, 42)	34 (28, 41)	0.159	0.14
RVSV, mL	88 (78, 101)	85 (72, 101)	0.359	0.11
RVSVi, mL/m^1.7^	46 (42, 53)	46 (39, 52)	0.252	0.02
RVEF, %	57 (53, 60)	57 (53, 61)	0.384	0.11
LAV maximal, mL	70 (58, 86)	75 (57, 90)	0.342	0.2
LAVi maximal, mL/m^1.7^	37 (30, 45)	39 (31, 47)	0.274	0.13
LAV minimal, mL	27 (20, 37)	30 (20, 41)	0.269	0.11
LAVi minimal, mL/m^1.7^	14 (11, 19)	16 (11, 21)	0.244	0.13
LASV, mL	42 (36, 50)	43 (34, 49)	0.868	0.09
LASVi, mL/m^1.7^	22 (19, 27)	22 (19, 26)	0.718	0.08
LAEF, %	61 (55, 66)	60 (53, 65)	0.291	0.1
RAV maximal, mL	80 (69, 101)	79 (69, 96)	0.843	0.06
RAVi maximal, mL/m^1.7^	43 (36, 53)	42 (35, 51)	0.846	0.05
RAV minimal, mL	42 (34, 56)	44 (35, 57)	0.519	0.07
RAVi minimal, mL/m^1.7^	22 (18, 29)	24 (18, 29)	0.455	0.11
RAEF, %	46 (42, 53)	44 (38, 50)	**0** **.** **018**	0.2
RASV, mL	38 (31, 46)	36 (28, 43)	0.082	0.21
RASVi, mL/m^1.7^	20 (16, 24)	19 (15, 23)	0.093	0.16
GCS, median (IQR)	−22 (−24, −19)	−22 (−25, −20)	0.438	0.1
GRS, median (IQR)	45 (39, 49)	44 (405, 51)	0.981	0.03
GLS, median (IQR)	−18 (−20, −16)	−18 (−20, −16)	0.709	0.09
Baseline medication				
Any Medications, %	140 (72.5)	140 (72.5)	1.00	<0.001
Beta blockers, %	34 (17.6)	53 (27.5)	**0** **.** **028**	0.22
ACEi, %	53 (27.5)	59 (30.6)	0.575	0.07
ARBi, %	33 (17.1)	42 (21.8)	0.303	0.12
Calcium blockers, %	48 (24.9)	58 (30.1)	0.305	0.01
Diuretics (Loop diuretics and thiazides), %	26 (13.5)	31 (16.1)	0.566	0.07
Nitrates, %	5 (2.6)	11 (5.7)	0.202	0.16
Antilipidemic agents, %	95 (49.2)	96 (49.7)	1.00	0.01
Anti-hyperglycaemic agents, %	18 (9.3)	17 (8.8)	1.00	0.02

CKD, chronic kidney disease; BMI, body mass index; IQR, interquartile range; LV, left ventricle; RV, right ventricle; LVEDV, left ventricular end-diastolic volume; LVEDVi, left ventricular end-diastolic volume indexed to height^1.7^; LVESV, left ventricular end-systolic volume; LVESVi, left ventricular end-systolic volume indexed to height^1.7^; LVEF, left ventricular ejection fraction; LVSV, left ventricular stroke volume; LVSVi, left ventricular stroke volume indexed to height^1.7^; LVCO, left ventricular cardiac output; LVCOi, left ventricular cardiac output indexed to height^1.7^; LVMi, left ventricular mass indexed to height^1.7^; WT, wall thickness; WTi, wall thickness indexed to height^1.7^; RVSV, right ventricular stroke volume; RVSVi, right ventricular stroke volume; RVEF, right ventricular ejection fraction; RVEDV, right ventricular end-diastolic volume; RVEDVi, right ventricular end-diastolic volume indexed to height^1.7^; RVESV, right ventricular end-systolic volume; RVESVi, right ventricular end-systolic volume indexed to height^1.7^; LAV, left atrial volume; LAVi, left atrial volume indexed to height^1.7^; LAEF, left atrial ejection fraction; LASV, left atrial stroke; Volume LASVi, left atrial stroke Volume indexed to height^1.7^; RAV, right atrial volume; RAVi, right atrial volume indexed to height^1.7^; RAEF, right atrial ejection fraction; GCS, global circumferential strain; GRS, global radial strain; GLS, global longitudinal strain.

Trend-level results are underlined. Statistically significant values are shown in bold.

### 4.3 Matched cohort analysis: imaging biomarkers & myocardial T1 differences

Following 1:1 matching, a total of 193 participants with CKD were matched to 193 non-CKD controls. The two groups were well balanced across demographic and clinical variables, with all standardized mean differences (SMDs) below conventional thresholds for imbalance (<0.1), (Table 1, Supplementary Figure S2).

#### 4.3.1 Demographics and clinical characteristics

There were no significant differences in age [median 68 (IQR: 62, 73) years, in both groups; *p* = 0.961], sex (57.5% female in both groups; *p* = 1.00), BMI [median 28 (25, 31) kg/m^2^ in both groups; *p* = 0.964], smoking status (2.1% in both groups; *p* = 1.00), or socioeconomic deprivation (Townsend index: −2.1 vs. −2.4; *p* = 0.775). Ethnic distributions were also well matched (*p* = 1.00), (Table 1).

#### 4.3.2 Comorbidities

Successful matching was achieved on key comorbidities, with identical prevalence of diabetes (17.6%), hypertension (70.5%), hyperlipidemia (57.5%), cardiac disease (32.1%), and stroke (6.7%) across groups (*p* = 1.00 for all comparisons; Table 1).

#### 4.3.3 Cardiac imaging biomarkers

Myocardial native T1 was trend level higher in CKD compared to non-CKD participants [median T1 = 929 (893, 958) ms vs. median 919 (889, 952) ms; *p* = 0.097]. CKD participants also demonstrated reduced LV cardiac output (5.2 vs. 5.4 L/min; *p* = 0.026) and cardiac output indexed to body surface area (2.7 vs. 2.9 L/min/m^2^; *p* = 0.006), (Table 1). No significant differences were observed in LV volumes, mass, or ejection fraction (e.g., LVEF: 59% in both groups; *p* = 0.649). RV measures were largely comparable, though trends toward lower RA stroke volume were observed in CKD, (Table 1).

#### 4.3.4 Atrial structure and function

Left and right atrial volumetric measures were similar across groups. However, RA ejection fraction was significantly reduced in the CKD group (44% vs. 46%; *p* = 0.018). Other atrial function measures did not differ significantly (Table 1).

#### 4.3.5 Medication use

Post-matching medication use was generally balanced. While the use of beta-blockers was higher among CKD participants (27.5% vs. 17.6%; *p* = 0.028), usage of other medication classes, were comparable with no significant differences (Table 1).

### 4.4 Twin study in non-CKD controls

In the control-to-control twin-matching methodology, the participant characteristics stratified by the non-CKD controls and their matched twins are presented in Supplementary Table S3. The results showed that the non-CKD control group was internally consistent, with minimal variability between matched individuals, making it a reliable reference for comparisons in the broader CKD-related analysis (Supplementary Table S4).

### 4.5 Differences in myocardial T1 differences in CKD cases and matched non-CKD controls

**Figure 2:**
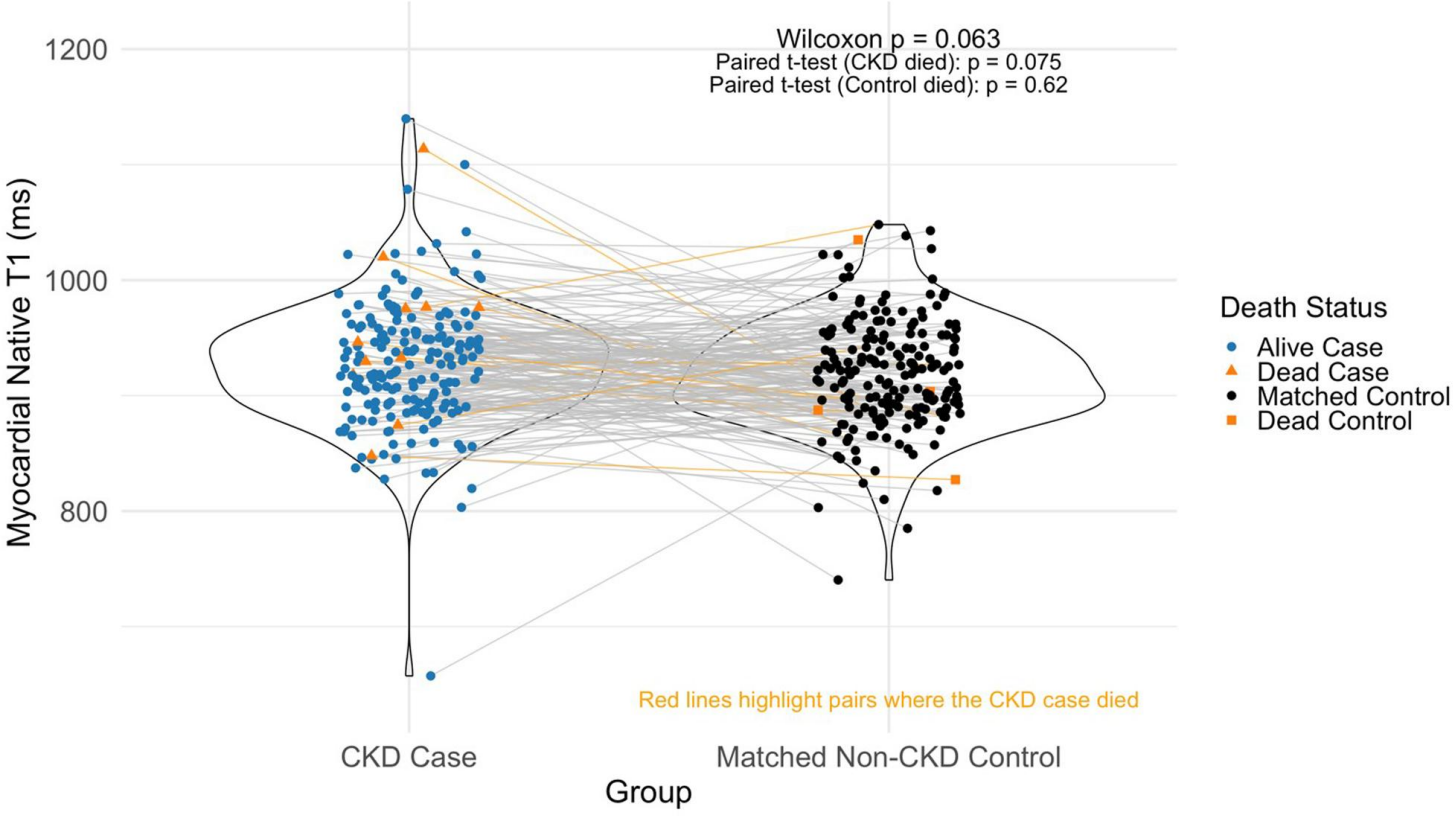
Paired Differences myocardial T1 values in CKD cases and their matched non-CKD controls across 193 matched pairs. Paired comparisons of myocardial native T1 values in 193 matched pairs. Each line connects a CKD case and its matched control.

In the full matched cohort (*n* = 193 pairs), CKD cases demonstrated a right-skewed distribution of native myocardial T1 values with a prominent upper tail, suggesting a greater burden of myocardial fibrosis and tissue abnormalities compared with non-CKD controls (Figure 2). In-pair T1 differences (CKD case minus control) were calculated for each matched pair. Among the 193 pairs, 108 (56%) showed higher T1 values in the CKD case than in the matched control. The range of in-pair differences spanned from −308 ms to +235 ms. The median paired difference was trend level significant [8 ms, interquartile range (IQR): −31, 46 ms], with a 95% CI: −1, 17 ms, *p* = 0.063) (Figure 2).

### 4.6 Outcome analysis

**Figure 3:**
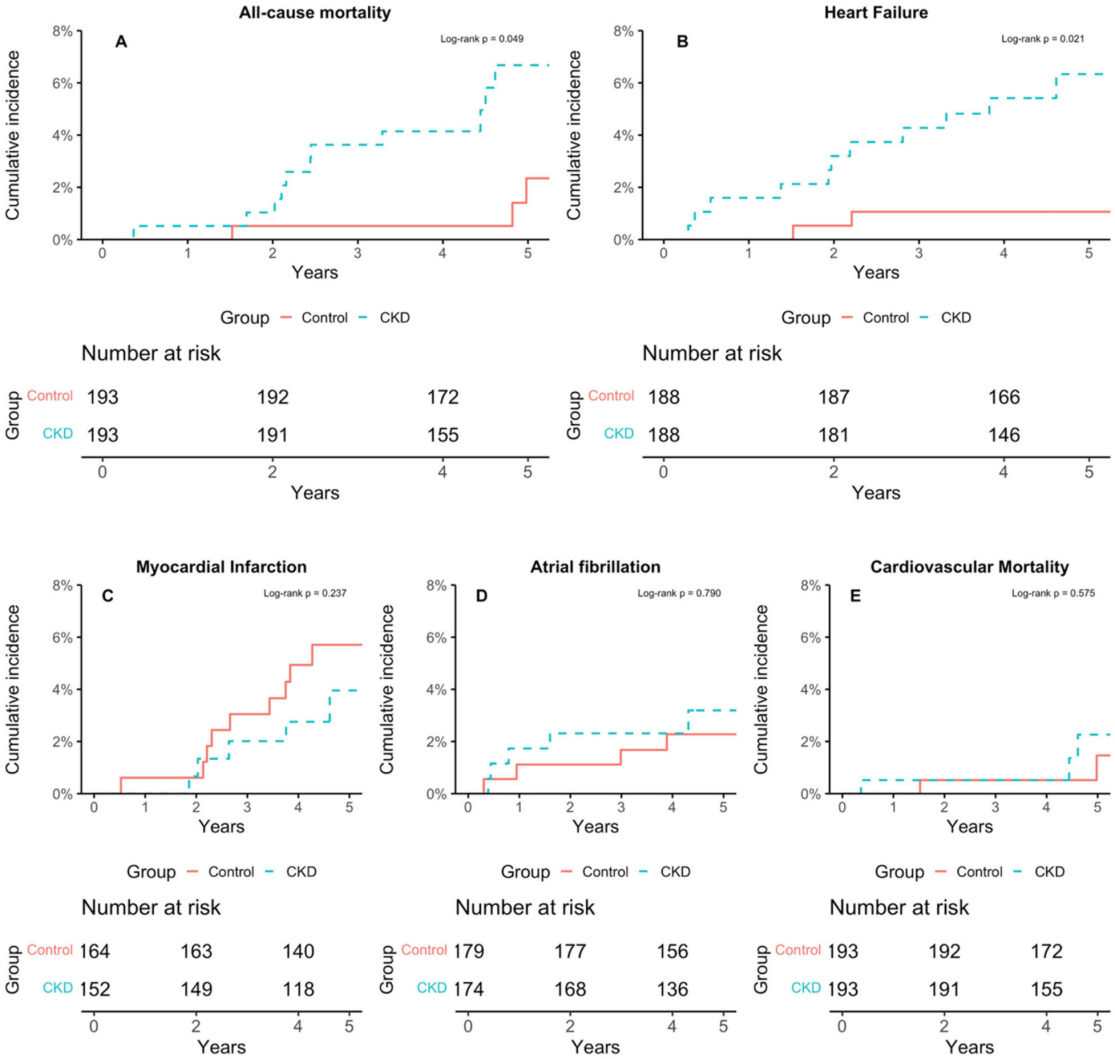
Cumulative incidence curves for incident outcomes in CKD cases and matched controls (truncated at median follow-up). Cumulative incidence curves compare CKD cases and matched non-CKD controls for (**A**) all-cause mortality, (**B**) heart failure, (**C**) myocardial infarction, (**D**) atrial fibrillation, and (**E**) cardiovascular mortality, to match the labels shown in the figure. Log-rank *p*-values indicate statistical differences between groups.

During a median follow-up of 4.9 years (IQR 4.2, 6.4), CKD cases had higher incidence rates of all-cause mortality [11.3 (95% CI 5.6, 20.1) vs. 3.8 (1.0, 9.7) per 1,000 person-years; *p* = 0.049] and heart failure [12.7 (6.7, 22.5) vs. 3.9 (1.06, 9.95) per 1,000 person-years; *p* = 0.021] than controls. Rates did not differ for cardiovascular death (3.07 vs. 1.88 per 1,000 person-years; log-rank *p* = 0.575), myocardial infarction (6.59 vs. 12.40; *p* = 0.237), or atrial fibrillation (6.92 vs. 6.18; *p* = 0.790) (Table 1; Figure 3).

#### 4.6.1 Myocardial T1 and all-cause and cardiovascular mortality in matched CKD cases and non-CKD controls

**Figure 4:**
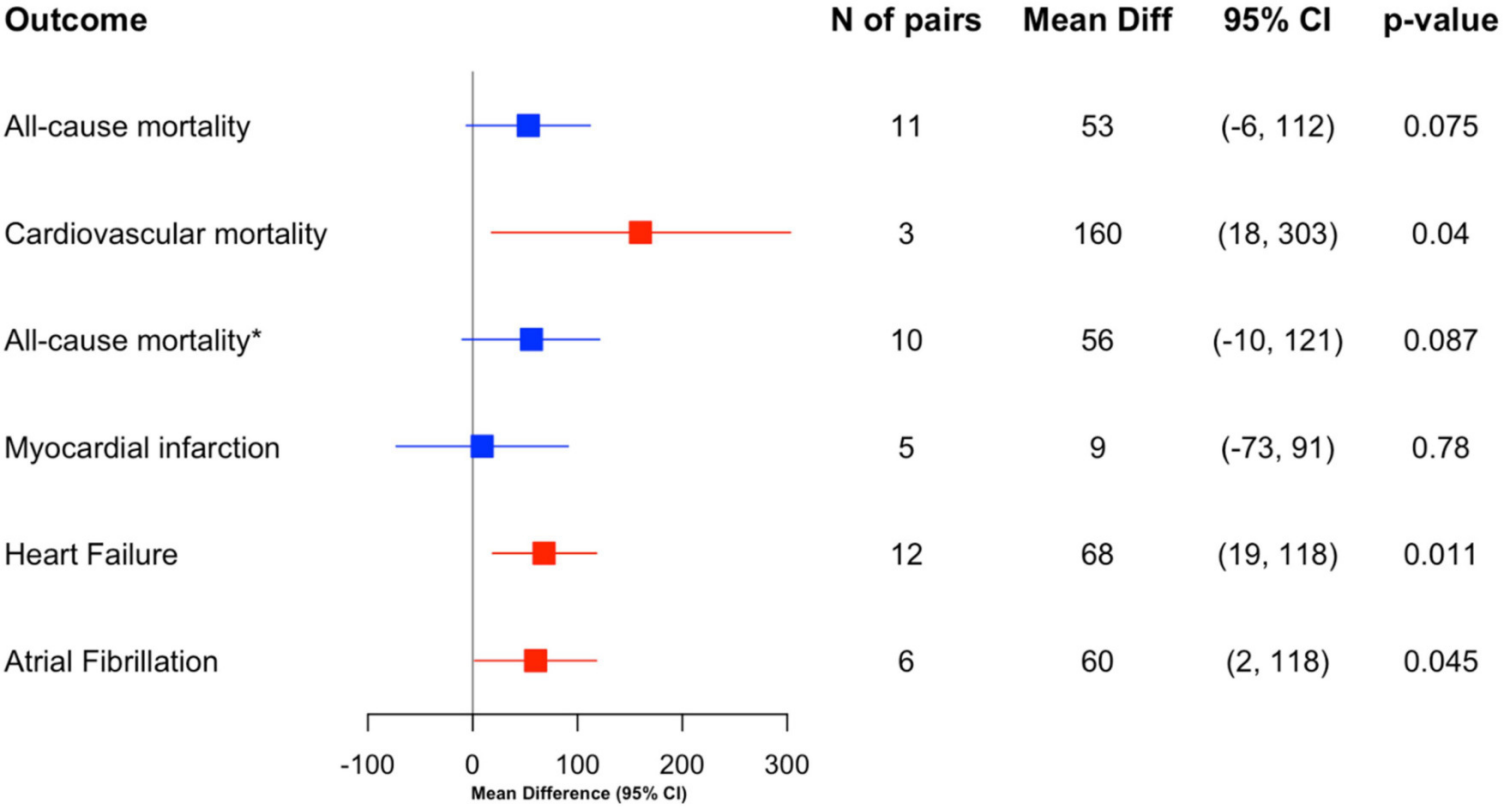
Forest plot of mean differences in myocardial T1 values in matched pairs categorized by the type of clinical outcomes. *Excludes 1 pair, where both CKD and control died. Each point represents the mean difference with 95% confidence intervals (CI) in T1 between CKD and matched control for each outcome. Statistically significant associations are indicated in red.

Among 11 matched pairs in which the CKD case died from any cause, the mean within-pair difference in native myocardial T1 was 53 ms (95% CI −6 to 112; SD 88; *p* = 0.075). Excluding the one pair in which both members died, the mean difference across 10 discordant pairs (CKD died, control survived) was 56 ms (95% CI −10 to 121; SD 92; *p* = 0.087). In the three pairs where the CKD case died of cardiovascular causes, the mean difference was 160 ms (95% CI 18 to 303; *p* = 0.040) (Table 2; Figure 4).

**Table 2:** Mean paired differences in T1 values for matched CKD participants and non-CKD controls with the specified clinical outcomes.

Outcome	N of pairs	Mean difference 95% CI	Mean difference SD	*P* value
All-cause mortality	11	53 (−6, 112)	88	0.075
Cardiovascular mortality	3	160 (18, 303)	58	**0** **.** **04**
All-cause mortality*	10	56 (−10, 121)	92	0.087
Myocardial infarction	5	9 (−73, 91)	66	0. 78
Heart Failure	12	68 (19, 118)	78	**0** **.** **011**
Atrial Fibrillation	6	60 (2, 118)	55	**0** **.** **045**

Trend-level results are underlined. Statistically significant values are shown in bold.

* Excludes 1 pair in which both the CKD case and the control died.

#### 4.6.2 Myocardial T1 and cardiovascular outcomes in matched CKD and non-CKD controls

For five pairs with incident myocardial infarction in the CKD case, the mean T1 difference was 9 ms (95% CI −73 to 91; SD 66; *p* = 0.78). In 12 pairs where the CKD case developed heart failure, native myocardial T1 was higher (mean difference 69 ms; 95% CI 19 to 118; SD 78; *p* = 0.011); no controls had heart failure. In six pairs with incident atrial fibrillation in the CKD case, T1 was higher (mean difference 60 ms; 95% CI 2 to 118; SD 55; *p* = 0.045); no controls had atrial fibrillation (Table 2).

#### 4.6.3 Reverse-pair analysis: myocardial T1 in non-CKD controls with adverse outcomes

In reverse-pair analyses, non-CKD controls with adverse outcomes generally had numerically higher T1 values for all-cause mortality and myocardial infarction, and lower T1 values for heart failure and atrial fibrillation, compared to CKD individuals who remained event-free; however, none of these differences were statistically significant (Supplementary Table S4).

#### 4.6.4 Associations between myocardial native T1 and outcomes

##### 4.6.4.1 CKD participants

**Figure 5:**
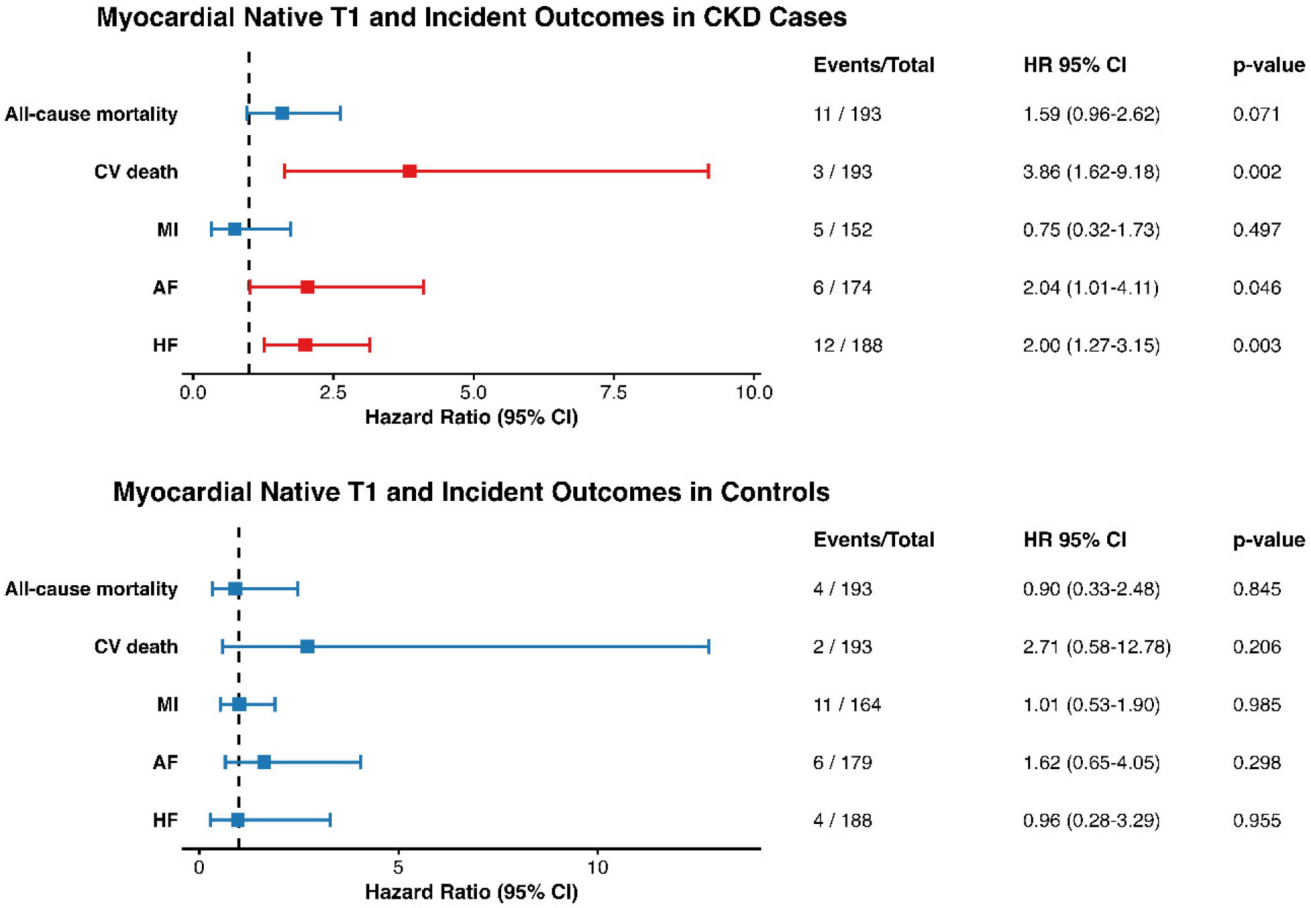
Associations of myocardial native T1 with incident outcomes in CKD cases and matched controls. Forest plots show hazard ratios (HR) and 95% confidence intervals (CI) for the association between myocardial native T1 and incident outcomes in CKD cases and matched controls. Outcomes include all-cause mortality, cardiovascular death, myocardial infarction (MI), atrial fibrillation (AF), and heart failure (HF). The table summarizes the number of events, total participants, HR (95% CI), and *p*-values for each outcome. Red indicates statistically significant associations (*p* < 0.05).

In participants with CKD, a 1-SD increase in native T1 was significantly associated with higher risk for cardiovascular death (HR: 3.86 (95% CI: 1.62 to 9.18; *p* = 0.002), atrial fibrillation: HR 2.04 (95% CI: 1.01 to 4.12; *p* = 0.046), and heart failure: HR: 2.00 (95% CI: 1.27 to 3.15; *p* = 0.003). Associations with all-cause mortality (HR: 1.59; 95% CI: 0.96 to 2.62; *p* = 0.071) and myocardial infarction (HR: 0.75; 95% CI: 0.32 to 1.73; *p* = 0.50) were not statistically significant (Figure 5; Table 3). Penalized Cox regression using Firth's correction was performed for endpoints with sparse events (cardiovascular death, atrial fibrillation, and myocardial infarction) and yielded estimates comparable in direction and magnitude to conventional Cox models (Supplementary Table S6). Competing-risk analysis for cardiovascular death produced similar results (HR: 3.9; 95% CI: 2.14 to 7.11).

**Table 3:** Associations of myocardial native T1 with incident outcomes in CKD cases and matched non-CKD controls.

Outcome	CKD cases			Non-CKD controls		
	Events/total	HR (95% CI)	*P*-value	Events/total	HR (95% CI)	*P*-value
All-cause mortality	11/193	1.59 (0.96, 2.62)	0.071	4/193	0.90 (0.33, 2.48)	0.845
Cardiovascular mortality	3/193	3.86 (1.62, 9.18)	**0** **.** **002**	2/193	2.72 (0.58, 12.78)	0.206
Myocardial infarction	5/152	0.75 (0.32, 1.73)	0.497	11/164	1.01 (0.53, 1.90)	0.985
Heart Failure	12/188	2.00 (1.27, 3.15)	**0** **.** **003**	4/188	0.97 (0.28, 3.29)	0.955
Atrial Fibrillation	6/174	2.04 (1.01, 4.12)	**0** **.** **046**	6/179	1.62 (0.65, 4.05)	0.296

Hazard ratios (HR) and 95% confidence intervals (CI) for the effect of a 1 standard deviation increase in myocardial native T1 on incident outcomes in CKD cases and matched non-CKD controls. Events/Total indicates the number of participants with the outcome and the total number included in each analysis. Trend-level results are underlined.

##### 4.6.4.2 Matched non-CKD controls

In non-CKD controls, T1 values were not significantly associated with any of the investigated outcomes, suggesting that the prognostic utility of T1 may be specific to CKD-related myocardial remodeling (Figure 5; Table 3).

##### 4.6.4.3 Secondary analysis

In stratified Cox models, the interaction between myocardial T1 and CKD status was not significant for any outcome (all *p* > 0.3), (Supplementary Table S5).

## 5 Discussion

We present, for the first time, the application of a prospective phenotypically identical twin study design to investigate rare diseases using pre-existing large-scale population data. Our methodology intentionally prioritized high phenotypic comparability: each CKD participant was matched 1:1 with a control exhibiting near-identical profiles across demographics, comorbidities, socioeconomic status, and cardiovascular risk factors. By focusing on carefully matched phenotypic comparators, this study demonstrates how twin-matching can enable precision-level epidemiologic inference in complex, multimorbid populations such as CKD.

While the data have been pre-acquired according to the UKBB protocols, the identification of cases was fully prospective and transparent. As a proof of principle, this method was applied to the UKBB database to investigate the relationship between baseline native myocardial T1 and tissue alterations-and incident cardiovascular outcomes in participants with CKD.

We report four main findings from this 1:1 virtual twin–matched cohort study. First, CKD participants had trend-level higher myocardial T1 values than controls. Second, CKD participants had higher cumulative incidences of all-cause mortality and heart failure compared to their matched controls, but no significant differences were observed for myocardial infarction, atrial fibrillation, or cardiovascular death. Third, myocardial native T1 values were consistently higher in CKD cases across outcome-specific paired analyses, with significant differences for cardiovascular death, heart failure, and atrial fibrillation. Fourth, in time-to-event Cox regression models, a 1-SD increase in myocardial T1 was associated with higher risks of cardiovascular death, atrial fibrillation, and heart failure in CKD participants, whereas no significant associations were observed in matched controls.

### 5.1 Myocardial T1 as a non-invasive biomarker of cardiac fibrosis in CKD

We found that CKD participants in UK Biobank had persistently elevated native myocardial T1 values compared to their matched non-CKD controls. These findings are consistent with earlier studies showing that native myocardial T1 correlates with markers of myocardial injury (hs-cTnT) and wall stress (NT-proBNP) in CKD (10). Unlike previous studies focused on highly selected or dialysis populations (6, 7), our study builds on this by including a broader CKD population-based cohort using a rigorous matching design to isolate myocardial signal attributable to kidney dysfunction.

### 5.2 Myocardial T1 and mortality outcomes in CKD

Elevated baseline native myocardial T1 values were significantly associated with cardiovascular mortality among CKD participants in both paired comparisons and even Cox models, suggesting that myocardial tissue abnormalities captured by native T1 reflect remodeling processes with diffuse myocardial disease as the driver of poor outcome. Although the association between myocardial T1 and all-cause mortality impact was just trend level, the direction and magnitude were consistent across analyses, and merit further investigation.

These findings align with previous studies linking elevated native T1 to increased risk of death in various clinical cohorts, extending this evidence into the CKD settings (12). Individuals with CKD experienced more than double the mortality risk of the general population, and over 50% of CKD-related deaths are due to cardiovascular causes (2), with this study pointing directly to the underlying mechanism in myocardial tissue properties detectable in non-invasive CMR scans. Mendelian randomization and observational data support a causal relationship between mild-to-moderate kidney dysfunction with cardiovascular outcomes such as coronary heart disease and sudden cardiac death (34, 35). While eGFR and albuminuria are established predictors of cardiovascular risk in CKD (16), they primarily reflect kidney impairment and systemic vascular injury; in contrast, native T1 mapping provides direct and quantitative non-invasive tissue characterization specific to the myocardium.

### 5.3 Myocardial T1 and incident cardiovascular outcomes

Elevated baseline native T1 was significantly associated with incident heart failure among CKD participants, both in paired comparisons and time-event models, supporting its role as a marker of myocardial fibrosis and remodeling in CKD-related cardiomyopathy. Heart failure is a major cardiovascular complication in CKD, affecting nearly 30% of CKD U.S. Medicare patients, as compared to just 6% in those without CKD (36). Risk rises with declining kidney function, as shown in the ARIC study, where individuals with an eGFR <60 mL/min/1.73 m^2^ had a threefold increase in heart failure risk (37). Echocardiography, while useful for detecting structural and functional abnormalities, is limited in its ability to characterize myocardial tissue alterations and detect focal and diffuse interstitial myocardial fibrosis (37). In the CRIC study, 50% of CKD patients without clinical signs of heart failure had left ventricular hypertrophy (38). In our study, T1 mapping identified early myocardial changes in CKD participants who later developed heart failure, preceding clinical manifestations, suggesting potential utility for pre-symptomatic risk stratification in this high-risk population.

In our study, elevated myocardial native T1 was significantly associated with a higher risk of incident atrial fibrillation among CKD participants, whereas no such association was observed in matched non-CKD controls. This finding suggests that myocardial tissue abnormalities detected by native T1 mapping may play a role in arrhythmogenic risk specifically within CKD populations. These findings extend previous epidemiological evidence showing that CKD and reduced eGFR are independent predictors of incident atrial fibrillation (39, 40). By leveraging myocardial tissue characterization, our study demonstrates that replacement fibrosis, quantified by elevated T1, is an important mechanistic pathway linking CKD to atrial fibrillation risk. This finding highlights the value of myocardial T1 mapping in refining arrhythmogenic risk prediction among CKD patients and suggests that structural myocardial remodeling, rather than kidney dysfunction alone, may drive AF development in this population.

### 5.4 Addressing methodological challenges in rare disease research

Prospective recruitment of patients with rare diseases together with closely matched controls is prohibitively cumbersome and expensive. Research involving less prevalent conditions—such as advanced CKD—within large population-based datasets is often challenged by sample size imbalances and confounding factors. Conventional multivariable regression approaches assume linear relationships and can perform poorly when case numbers are limited, potentially introducing bias and overfitting. Moreover, while large datasets like the UK Biobank offer statistical power, they may also introduce sampling heterogeneity and potentially erroneous finding that may arise from poor matching between groups (41). Basic statistical methods can address confounders, but the commonly-used linear adjustment models are often influenced by the larger control groups, and may be misapplied to the smaller, targeted disease groups (41).

To address these limitations, we applied an artificial twin-matching strategy to construct well-balanced 1:1 matched cohort of CKD and non-CKD participants. This method enables tight control over demographic, clinical, cardiovascular, and socioeconomic variables, thereby enhancing the internal validity of our comparisons and preserving biological signals. Given the large volume of UK Biobank datasets, we were able to perform matching to minimize baseline imbalances improving the reliability of T1 comparisons, without the need to involve complex statistical modelling for confounds. The paired within-twin analyses allowed us to explore non-linear differences in myocardial T1 across outcomes by comparing each CKD participant directly with their matched control. In contrast, the stratified Cox models quantified the linear associations between myocardial T1 and time-to-event outcomes, expressed per 1-SD increase in T1. The complementary findings from these two approaches strengthen the robustness of our conclusions, demonstrating that myocardial fibrosis, as reflected by elevated T1, is strongly linked to adverse outcomes in CKD.

### 5.5 Study limitations and future directions

Several limitations should be acknowledged. In this study, CKD was defined based on ICD-10 diagnosis codes recorded prior to imaging. This likely captured predominantly individuals with more advanced or clinically recognized CKD, rather than early-stage or undiagnosed disease. As a result, the observed prevalence in the UK Biobank imaging cohort was ∼1%, which was substantially lower than the estimated global prevalence of ∼10% for any-stage CKD (1). This discrepancy likely reflects under-ascertainment due to coding-based identification and the healthy volunteer bias of the UK Biobank cohort.

The number of events for certain outcomes—particularly cardiovascular mortality—was limited, resulting in reduced statistical power and increased model instability. Although penalized Cox and competing-risk analyses were applied to mitigate small-sample bias, the sparse event counts restrict the precision and reliability of these estimates. Accordingly, findings for these endpoints should be interpreted cautiously and considered hypothesis-generating, warranting validation in larger imaging cohorts. An additional consideration is T1 quality control. In the UK Biobank quality-control study of automated T1 processing, the comparison between visually quality-checked maps and automatically processed T1 maps was performed only in participants without overt cardiovascular disease (42). In contrast, in our study, over 30% of participants had pre-existing cardiovascular disease at baseline, a context where T1 artefacts may be more prevalent. We therefore carefully examined extreme T1 values, noting that low outlier values in CKD are likely artefact-related and may require exclusion or targeted data recovery after individual quality review. Given the single-cohort design and potential selection related to CMR/T1 availability, validation in larger and independent cohorts is needed. Looking forward, we aim to expand the CKD cohort, incorporate manual quality control of T1 data to recover cases rejected by automated QC (Dice score <0.7), and integrate longitudinal biomarkers and imaging data. These enhancements will improve phenotypic precision and support the development of myocardial T1 as a clinically useful, non-invasive biomarker for cardiovascular risk stratification in CKD.

## 6 Clinical and research implications

Our findings support the link between myocardial tissue properties, as measured by native T1 mapping, for identifying myocardial remodeling in CKD, in relation to heart failure, atrial fibrillation and cardiovascular mortality. This study supports the integration of T1 mapping into clinical assessment algorithms to improve early and more accurate cardiovascular risk stratification in CKD, beyond conventional metrics such as left ventricular mass. As a much wider implication, we demonstrated here a general method to utilize the enormous diligence and volumes of data acquired in modern national large-scale population databases. The proposed identical twin study design mimics truthfully an outcome of a prospective clinical study design. While the data were pre-acquired, the selection criteria, based on open information in the public database, are fully prospective. This improves transparency and results in much larger achievable sample sizes in rare diseases, with potential bootstrapping, beyond the single additional double-control group demonstrated in this study.

## 7 Conclusions

Among CKD participants, elevated native myocardial T1was independently associated with increased risks of heart failure, cardiovascular mortality, and atrial fibrillation. By applying a virtual twin-matching framework based on multidimensional phenotypic similarity, this study maximized comparability between CKD cases and controls and improved internal validity. Our approach demonstrates the potential of precision-matched cohort designs to enhance mechanistic inference and risk stratification in complex, multimorbid populations.

## Data Availability

This research was conducted using the UK Biobank resource under approved application [Application Number 59867]. The tabular data used in this study are available to bona fide researchers upon successful application to UK Biobank. Due to data governance and participant confidentiality agreements, the dataset cannot be shared publicly by the authors. Requests to access these datasets should be directed to www.ukbiobank.ac.uk.
