# Supplementary material for: Myocardial fibrosis and tissue alterations predict cardiovascular outcomes in chronic kidney disease—a prospective virtual twin study design using large-scale population database
Source: Front Cardiovasc Med. 2026 May 11;13:1726445. doi: 10.3389/fcvm.2026.1726445 (PMC13199255; doi:10.3389/fcvm.2026.1726445)
Supplement: Supplementary file 1 [file Supplementaryfile1.docx]

**Supplementary Material**

**Myocardial Fibrosis and Tissue Alterations Predict Cardiovascular Outcomes in Chronic Kidney Disease – A prospective virtual twin study design using large scale**

**population database**

**Authors:** Georgios Vavilis^1^, Yeshe M. Kway, Zahra Raisi-Estabragh^3,4^, Steffen E Petersen^3,4,5^, Stefan Neubauer^1^, Qiang Zhang^1,2^, Vanessa M Ferreira*^1^, Stefan K Piechnik*^1^

^1^Division of Cardiovascular Medicine, Oxford Centre for Clinical Magnetic Resonance Research, Radcliffe Department of Medicine, University of Oxford;

^2^National Institute for Health Research Oxford Biomedical Research Centre, Oxford University Hospitals NHS Foundation Trust, Oxford OX3 9DU, UK;

^3^Barts Heart Centre, St Bartholomew’s Hospital, Barts Health NHS Trust, West Smithfield, London EC1A 7BE, UK;

^4^William Harvey Research Institute, NIHR Barts Biomedical Research Centre, Queen Mary University of London, Charterhouse Square, London EC1M 6BQ, UK;

^5^Health Data Research UK, London NW1 2BE, UK Department of Medicine, Huddinge; Heart and Vascular Theme, Division of Coronary and Valvular Heart Disease, Karolinska University Hospital, Stockholm, Sweden.

* Vanessa M Ferreira, Stefan K Piechnik contributed equally to this work

Table of Contents

[Supplementary Table 1. Disease definitions 3](#_Toc222745850)

[Supplementary Table 2: Baseline Characteristics before matching 13](#_Toc222745851)

[Supplementary table 3: Baseline Characteristics of matched twins and their non-CKD controls 17](#_Toc222745852)

[Supplementary table 4. Reverse-Pair Analysis: Myocardial T1 in Non-CKD Controls with Adverse Outcomes 21](#_Toc222745853)

[Supplementary Table 5. Associations Between Myocardial Native T1, CKD Status, and Incident Outcomes Using Stratified Cox Regression Models 22](#_Toc222745854)

[Supplementary Table 6. Associations of Myocardial Native T1 with Incident Outcomes in CKD Cases and Matched Non-CKD Controls: Results from Penalised Cox Regression 23](#_Toc222745855)

[Supplementary figures 24](#_Toc222745856)

[Supplementary Figure 1. The Covariate Balance plot: Comparison of the balance of covariates between CKD cases and control groups before and after matching. 24](#_Toc222745857)

# **Supplementary Table 1. Disease definitions**

| **Source** | **UKB Field ID / Code** | **Description** |
| --- | --- | --- |
| **Cerebrovascular diseases** | | |
| ICD10 | I65 | Occlusion and stenosis of precerebral arteries, not resulting in cerebral infarction |
|  | I66 | Occlusion and stenosis of cerebral arteries, not resulting in cerebral infarct |
|  | I67 | Other cerebrovascular diseases |
|  | I68 | Cerebrovascular disorders in diseases classified elsewhere |
| First occurrences | 131370 | Date I65 first reported (occlusion and stenosis of precerebral arteries, not resulting in cerebral infarction) |
|  | 131372 | Date I66 first reported (occlusion and stenosis of cerebral arteries, not resulting in cerebral infarction) |
|  | 131374 | Date I67 first reported (other cerebrovascular diseases) |
|  | 131376 | Date I68 first reported (cerebrovascular disorders in diseases classified elsewhere) |
| **Neurological injury/trauma** | | |
|  | 131372 | Date I66 first reported (occlusion and stenosis of cerebral arteries, not resulting in cerebral infarction) |
|  | 131374 | Date I67 first reported (other cerebrovascular diseases) |
|  | 131376 | Date I68 first reported (cerebrovascular disorders in diseases classified elsewhere) |
|  | 131372 | Date I66 first reported (occlusion and stenosis of cerebral arteries, not resulting in cerebral infarction) |
| **Subarachnoid haemorrhage** | | |
| Self-report | 20002 | Subarachnoid haemorrhage |
| ICD9 | 430 | Subarachnoid haemorrhage |
| ICD10 | I60 | Subarachnoid haemorrhage |
| First occurrences | 131360 | Subarachnoid haemorrhage |
| Algorithm | 42012 | Date of subarachnoid haemorrhage (should be covered by 42006) |
| **Transient ischaemic attack (TIA)** | | |
| Self-report | 20002 | Transient ischaemic attack (TIA) |
| ICD9 | 435 | Transient cerebral ischaemia |
| ICD10 | G45 | Transient cerebral ischaemic attacks and related syndromes |
| First occurrences | 131056 | Transient cerebral ischaemic attacks and related syndromes |
| **Stroke** |  |  |
| Self-report | 20002 | Stroke |
|  | 20002 | Ischaemic stroke |
|  | 20002 | Brain haemorrhage |
| ICD9 | 431 | Intracerebral haemorrhage |
|  | 432 | Other and unspecified intracranial haemorrhage |
| ICD10 | I64 | Stroke, not specified as haemorrhage or infarction |
|  | I63 | Cerebral infarction |
|  | I61 | Intracerebral haemorrhage |
|  | I62 | Other nontraumatic intracranial haemorrhage |
| First occurrences | 131368 | Date I64 first reported (stroke, not specified as haemorrhage or infarction) |
|  | 131366 | Cerebral infarction |
|  | 131362 | Intracerebral haemorrhage |
|  | 131364 | Other nontraumatic intracranial haemorrhage |
| Diagnosed by doctor | 4056 | Age stroke diagnosed |
|  | 6150: 3 | Stroke |
| Algorithm | 42006 | Date of stroke |
|  | 42008 | Date of ischaemic stroke |
|  | 42010 | Date of intracerebral haemorrhage |
| **Cardiac arrhythmia** | | |
| Self-report | 20002 | Sick sinus syndrome |
|  | 20002 | SVT / supraventricular tachycardia |
|  | 20002 | Atrial flutter |
|  | 20002 | Heart arrhythmia |
|  | 20002 | Irregular heartbeat |
| ICD10 | I44.1 | Atrioventricular block, second degree |
|  | I44.2 | Atrioventricular block, complete |
|  | I45.3 | Tri fascicular block |
|  | I45.6 | Preexcitation syndrome |
|  | I46.0 | Cardiac arrest with successful resuscitation |
|  | I46.1 | Sudden cardiac death, so described |
|  | I46.9 | Cardiac arrest, unspecified |
|  | I47.0 | Re-entry ventricular arrhythmia |
|  | I47.1 | Supraventricular tachycardia |
|  | I47.2 | Ventricular tachycardia |
|  | I47.9 | Paroxysmal tachycardia, unspecified |
|  | I48.3 | Typical atrial flutter |
|  | I48.4 | Atypical atrial flutter |
|  | I49.0 | Ventricular fibrillation and flutter |
|  | I49.5 | Sick sinus syndrome |
| First occurrences | 131346 | Cardiac arrest |
|  | 131348 | Paroxysmal tachycardia |
|  | 131350 | Atrial fibrillation and flutter |
| **Cardiac arrhythmia (Atrial fibrillation)** | | |
| Self-report | 20002 | Atrial fibrillation |
| ICD10 | I48.0 | Paroxysmal atrial fibrillation |
|  | I48.1 | Persistent atrial fibrillation |
|  | I48.2 | Chronic atrial fibrillation |
|  | I48.9 | Atrial fibrillation and atrial flutter, unspecified |
| **Heart failure (unspecified aetiology)** | | |
| Self-report | 20002 | Heart failure/pulmonary oedema |
| ICD10 | I50.0 | Congestive heart failure |
|  | I50.1 | Left ventricular failure |
|  | I50.9 | Heart failure, unspecified |
| First occurrences | 131354 | Heart failure |
| **Ischaemic heart disease** | | |
| Self-report | 20002 | Angina |
| ICD10 | I20 | Angina pectoris |
|  | I24 | Other acute ischaemic heart diseases |
|  | I25 | Chronic ischaemic heart disease |
| First occurrences | 131296 | Angina pectoris |
|  | 131304 | Other acute ischaemic heart diseases |
|  | 131306 | Chronic ischaemic heart disease |
| Diagnosed by doctor | 3627 | Age angina diagnosed |
|  | 6150: 2 | Angina |
| **Ischaemic heart disease (Myocardial infarction)** | | |
| Self-report | 20002 | Heart attack/myocardial infarction |
| ICD9 | 410 | Acute myocardial infarction |
|  | 411 | Other acute and subacute forms of ischaemic heart disease |
|  | 412 | Old myocardial infarction |
| ICD10 | I21 | Acute myocardial infarction |
|  | I22 | Subsequent myocardial infarction |
|  | I23 | Certain current complications following acute myocardial infarction |
| First occurrences | 131298 | Acute myocardial infarction |
|  | 131300 | Subsequent myocardial infarction |
|  | 131302 | Certain current complications following acute myocardial infarction |
| Diagnosed by doctor | 3894 | Age heart attack diagnosed |
|  | 6150: 1 | Heart attack |
| Algorithm | 42000 | Date of myocardial infarction |
| **Valvular heart disease** | | |
| Self-report | 20002 | Mitral stenosis |
|  | 20002 | Mitral valve disease |
|  | 20002 | Heart valve problem/heart murmur |
|  | 20002 | Mitral regurgitation / incompetence |
|  | 20002 | Aortic valve disease |
|  | 20002 | Aortic stenosis |
|  | 20002 | Aortic regurgitation / incompetence |
| ICD10 | I34.0 | Mitral (valve) insufficiency |
|  | I34.2 | Non-rheumatic mitral (valve) stenosis |
|  | I34.8 | Other nonrheumatic mitral valve disorders |
|  | I34.9 | Non-rheumatic mitral valve disorder, unspecified |
|  | I35 | Non-rheumatic aortic valve disorders |
|  | I36 | Non-rheumatic tricuspid valve disorders |
|  | I37 | Pulmonary valve disorders |
|  | I38 | Endocarditis, valve unspecified |
|  | I39.0 | Mitral valve disorders in diseases classified elsewhere |
|  | I39.1 | Aortic valve disorders in diseases classified elsewhere |
|  | I39.3 | Pulmonary valve disorders in diseases classified elsewhere |
|  | I39.4 | Multiple valve disorders in diseases classified elsewhere |
|  | I39.8 | Endocarditis, valve unspecified, in diseases classified elsewhere |
|  | I05 | Rheumatic mitral valve diseases |
|  | I06 | Rheumatic aortic valve diseases |
|  | I07 | Rheumatic tricuspid valve diseases |
|  | I08 | Multiple valve diseases |
| First occurrences | 131322 | Non-rheumatic mitral valve disorders |
|  | 131324 | Non-rheumatic aortic valve disorders |
|  | 131326 | Non-rheumatic tricuspid valve disorders |
|  | 131328 | Pulmonary valve disorders |
|  | 131330 | Endocarditis, valve unspecified |
|  | 131332 | Endocarditis and heart valve disorders in diseases classified elsewhere |
|  | 131276 | Rheumatic mitral valve diseases |
|  | 131278 | Rheumatic aortic valve diseases |
|  | 131280 | Rheumatic tricuspid valve diseases |
|  | 131282 | Multiple valve diseases |
| **Chronic Kidney Disease** | | |
| Self-report | 20004 | Renal/kidney transplant |
|  | 20004 | Dialysis access surgery |
|  | 20004 | Haemodialysis access/fistula surgery |
|  | 20004 | Peritoneal dialysis (capd) access surgery |
|  | 20002 | Renal failure requiring dialysis |
|  | 20004 | Renal/kidney transplant |
|  | 20004 | Dialysis access surgery |
| OPCS4 |  | Insertion of arteriovenous prosthesis |
|  |  | Creation of arteriovenous fistula NEC |
|  |  | Attention to arteriovenous shunt |
|  |  | Banding of arteriovenous fistula |
|  |  | Thrombectomy of arteriovenous fistula |
|  |  | Creation of graft fistula for dialysis |
|  |  | Other specified arteriovenous shunt |
|  |  | Unspecified arteriovenous shunt |
|  |  | Allotransplantation of kidney from live donor |
|  |  | Allotransplantation of kidney from cadaver NEC |
|  |  | Allotransplantation of kidney from cadaver heart beating |
|  |  | Allotransplantation of kidney from cadaver heart non-beating |
|  |  | Other specified transplantation of kidney |
|  |  | Unspecified transplantation of kidney |
|  |  | Exploration of transplanted kidney |
|  |  | Pre-transplantation of kidney work-up – recipient |
|  |  | Post-transplantation of kidney examination – recipient |
|  |  | Other specified interventions associated with transplantation of kidney |
|  |  | Unspecified interventions associated with transplantation of kidney |
|  |  | Renal dialysis |
|  |  | Peritoneal dialysis NEC |
|  |  | Haemodialysis NEC |
|  |  | Hemofiltration |
|  |  | Automated peritoneal dialysis |
|  |  | Continuous ambulatory peritoneal dialysis |
|  |  | Hemoperfusion |
|  |  | Other specified compensation for renal failure |
|  |  | Unspecified compensation for renal failure |
|  |  | Insertion of ambulatory peritoneal dialysis catheter |
|  |  | Removal of ambulatory peritoneal dialysis catheter |
|  |  | Other specified placement of ambulatory apparatus for compensation for renal failure |
|  |  | Unspecified placement of ambulatory apparatus for compensation for renal failure |
|  |  | Insertion of temporary peritoneal dialysis catheter |
|  |  | Other specified placement of other apparatus for compensation for renal failure |
|  |  | Unspecified placement of other apparatus for compensation for renal failure |
|  |  | Insertion of arteriovenous prosthesis |
| ICD9 | 585 | Chronic renal failure |
|  | 5859 | Chronic renal failure |
| ICD10 | N165 | Renal tubulo-interstitial disorders in transplant rejection |
|  | N180 | N18.0 End-stage renal disease |
|  | N183 | N18.3 Chronic kidney disease, stage 3 |
|  | N184 | N18.4 Chronic kidney disease, stage 4 |
|  | N185 | N18.5 Chronic kidney disease, stage 5 |
|  | N188 | N18.8 Other chronic renal failure |
|  | N189 | N18.9 Chronic renal failure, unspecified |
|  | T824 | Mechanical complication of vascular dialysis catheter |
|  | T861 | Kidney transplant failure and rejection |
|  | Y602 | Unintentional cut, puncture, perforation or haemorrhage during surgical and medical care - During kidney dialysis or other perfusion |
|  | Y612 | Foreign object accidentally left in body during surgical and medical care - During kidney dialysis or other perfusion |
|  | Y622 | Failure of sterile precautions during surgical and medical care - During kidney dialysis or other perfusion |
|  | Y841 | Other medical procedures as the cause of abnormal reaction of the patient, or of later complication, without mention of misadventure at the time of the procedure - Kidney dialysis |
|  | Z490 | Preparatory care for dialysis |
|  | Z491 | Extracorporeal dialysis |
|  | Z492 | Other dialysis |
|  | Z940 | Kidney transplant status |
|  | Z992 | Dependence on renal dialysis |
|  | N165 | Renal tubulo-interstitial disorders in transplant rejection |
|  | T861 | Kidney transplant failure and rejection |
| **Hypertension** | | |
| Self-report | 20002 | Hypertension |
|  | 20002 | Essential hypertension |
| Medications | 6153 | Blood pressure medication |
| ICD10 | I10 | Essential (primary) hypertension |
| Diagnosed by doctor | 6150 | Age high blood pressure diagnosed |
|  | 6152 | High blood pressure |
| Hyperlipidaemia | | |
| Self-report | 20002 | High cholesterol |
| Medications | 6153 | Cholesterol lowering medication |
|  | 6177 |  |
| ICD10 | I10 | Essential (primary) hypertension |
| Diagnosed by doctor | 6150 | Age high blood pressure diagnosed |
|  | 6152 | High blood pressure |
|  | E780 | Pure hypercholesterolaemia |
|  | E782 | Mixed hyperlipidaemia |
|  | E783 | Hyperchylomicronaemia |
|  | E784 | Other hyperlipidaemia |
|  | E785 | Hyperlipidaemia, unspecified |
| First occurrences | 130814 | Disorders of lipoprotein metabolism and other lipidaemia |
|  | 131286 | first reported (essential (primary) hypertension) |
| Medications | 20003 | Simvastatin |
|  |  | Fluvastatin |
|  |  | Pravastatin |
|  |  | Atorvastatin |
|  |  | Rosuvastatin |
| **Diabetes** | | |
| Self-reported | 20002 | Diabetes |
|  |  | Type 1 diabetes |
|  |  | Type 2 diabetes |
| Medications | 6153 | Insulin |
|  | 6177 |  |
| ICD9 | 250 | Diabetes mellitus |
| ICD10 | E10 | Type 1 diabetes mellitus |
|  | E11 | Type 2 diabetes mellitus |
|  | E13 | Other specified diabetes mellitus |
|  | E14 | Unspecified diabetes mellitus |
|  | G590 | Diabetic mononeuropathy |
|  | G632 | Diabetic polyneuropathy |
|  | H280 | Diabetic cataract |
|  | H360 | Diabetic retinopathy |
|  | M142 | Diabetic arthropathy |
|  | N083 | Glomerular disorders in diabetes mellitus |
|  | O240 | Diabetes mellitus in pregnancy: Pre-existing type 1 diabetes mellitus |
|  | O241 | Diabetes mellitus in pregnancy: Pre-existing type 2 diabetes mellitus |
| First occurrences | 130706 | First reported (insulin-dependent diabetes mellitus) |
|  | 130708 | First reported (non-insulin-dependent diabetes mellitus) |
|  | 130712 | First reported (other specified diabetes mellitus) |
|  | 130714 | First reported (unspecified diabetes mellitus) |
| Diagnosed by doctor | 2443 | Diabetes diagnosed by doctor |
|  | 2976 | Age diabetes diagnosed by doctor |
|  | 2986 | Insulin within one year of diagnosis |
| Medications | 6153 | Insulin |
|  | 6177 |  |
| Medications | 20003 | Insulin product |

# **Supplementary Table 2:** **Baseline Characteristics before matching**

| **Covariates** | **Overall**  **N = 29233** | **Non-CKD**  **N = 29032** | **CKD**  **N= 201** | **P-value** |
| --- | --- | --- | --- | --- |
|  |  |  |  |  |
| Sex, % | 14058 (48.1) | 13944 (48.0) | 114 (56.7) | **0.017** |
| Age | 64 (57, 69) | 64 (57, 69) | 67 (62, 73) | **<0.001** |
| BMI, Kg/m^2^ | 26 (24, 29) | 26 (24, 29) | 28 (25, 32) | **<0.001** |
| Smoker, % | 1078 (3.7) | 1073 (3.7) | 5 (2.5) | 0.473 |
| Ethnic Q-risk, % |  |  |  | **<0.001** |
| 1 | 28435 (97.3%) | 28246 (97.3%) | 189 (94.0%) |  |
| 2 | 194 (0.7%) | 192 (0.7%) | 2 (1.0%) |  |
| 3 | 57 (0.2%) | 57 (0.2%) | 0 (0.0%) |  |
| 4 | 7 (0.0%) | 7 (0.0%) | 0 (0.0%) |  |
| 5 | 99 (0.3%) | 99 (0.3%) | 0 (0.0%) |  |
| 6 | 99 (0.3%) | 99 (0.3%) | 0 (0.0%) |  |
| 7 | 74 (0.3%) | 74 (0.3%) | 0 (0.0%) |  |
| 8 | 88 (0.3%) | 88 (0.3%) | 0 (0.0%) |  |
| 9 | 238 (0.8%) | 238 (0.8) | 10 (5.0%) |  |
| Deprivation Townsend, | -2.67 (-3.93, -0.66) | -2.68 (-3.93, -0.66) | -2.01 (-3.75, 0.27) | **0.012** |
| Diabetes, % | 1636 (5.6) | 1595 (5.5) | 41 (20.4) | **<0.001** |
| Hypertension, % | 9595 (32.8) | 9452 (32.6) | 143 (71.1) | **<0.001** |
| Hyperlipidaemia, % | 8336 (28.5) | 8218 (28.3) | 118 (58.7) | **<0.001** |
| Any baseline known cardiac disease, % | 3424 (11.7) | 3357 (11.6) | 67 (33.3) | **<0.001** |
| Stroke, % | 629 (2.2) | 614 (2.1) | 15 (7.5) | **<0.001** |
| Native Myocardial T1, ms | 923 (894, 953) | 923 (894, 953) | 929 (893, 958) | 0.345 |
| LVEDV, mL | 144.12 (124, 169) | 144(124, 169) | 147 (122, 169) | 0.764 |
| LVESV, mL | 58(47, 71) | 58 (47, 71) | 58 (47, 76) | 0.712 |
| LVSV, mL | 86 (74, 100) | 86 (74, 100) | 86 (73, 100) | 0.667 |
| LVEF, % | 60 (56, 64) | 60 (56, 64) | 59 (55, 64) | 0.454 |
| LVCO, L/min | 5.3 (4.6, 6.2) | 5.3 (4.6, 6.2) | 5.2 (4.3, 6.0) | 0.050 |
| LVM, g | 83 (69, 101) | 83 (69, 101) | 89 (71, 103) | **0.042** |
| WT, mm | 5.6 (5.1, 6.2) | 5.6 (5.1, 6.2) | 5.8 (5.4, 6.5) | **0.001** |
| LVEDVi, mL/m^1.7^ | 78 (70, 87) | 78 (70, 87) | 77 (67, 86) | 0.087 |
| LVESVi, mL/m^1.7^ | 31 (27, 37) | 31 (27, 37) | 30 (26, 37) | 0.314 |
| LVSVi, ml | 47 (41, 52) | 47 (41, 52) | 45 (40, 50) | **0.016** |
| LVCOi, L/min/m^1.7^ | 2.9 (2.5, 3.2) | 2.9 (2.5, 3.2) | 2.7 (2.4, 3.1) | **0.001** |
| LVMi, g/m^1.7^ | 45 (40, 51) | 45 (40, 51) | 46 (40, 53) | 0.280 |
| WTi, mm/m^1.7^ | 3.00 (2.8, 3.3) | 3.0 (2.8, 3.3) | 3.1 (2.8, 3.3) | 0.202 |
| Max WTi, mm/m^1.7^ | 3.8 (3.5 4.1) | 3.8 (3.5, 4.1) | 3.8 (3.6, 4.1) | 0.174 |
| Max WT, gr | 7.00 (6.3, 7.7) | 7.00 (6.3, 8.00) | 7.10 (6.5, 8) | **0.002** |
| RVEDV, mL | 152 (129, 180) | 152 (129, 180) | 155 (126, 176) | 0.586 |
| RVESV, mL | 65 (52, 80) | 65 (52, 81) | 64 (52, 79) | 0.627 |
| RVSV, mL | 87 (75, 102) | 87 (75, 102) | 85(72, 100) | 0.364 |
| RVEF, % | 58 (54, 61) | 58 (54, 61) | 57 (53, 62) | 0.965 |
| RVEDVi, mL/m^1.7^ | 82 (73, 93) | 82 (73, 93) | 81 (69, 91) | **0.025** |
| RVESVi, mL/m^1.7^ | 35 (29, 41) | 35 (29, 41) | 34 (28, 41) | 0.094 |
| RVSVi, mL/m^1.7^ | 47 (42, 53) | 47 (42, 53) | 45 (39, 52) | **0.007** |
| Max LAV, mL | 70 (57, 85) | 70 (57, 85) | 75 (57, 90) | 0.067 |
| Min LAV, mL | 27 (20, 35) | 27 (20, 35) | 31 (20, 41) | **0.013** |
| LASV, mL | 43 (35, 51) | 43 (35, 51) | 43 (34, 49) | 0.479 |
| LAEF, % | 61 (56, 66) | 61 (56, 66) | 60 (53, 65) | **0.002** |
| Max LAVi, mL/m^1.7^ | 38 (31, 45) | 38 (31, 45) | 39 (32, 47) | 0.320 |
| Min LAVi, mL/m^1.7^ | 15 (11, 19) | 15 (11, 19) | 16 (11, 21) | **0.040** |
| LASVi, mL/m^1.7^ | 23 (20, 27) | 23 (20, 27) | 22 (19, 26) | **0.031** |
| Max RAV, mL | 82 (68, 100) | 82 (68, 100) | 78 (67, 96) | 0.374 |
| Min RAV, mL | 43 (34, 55) | 43 (34, 55) | 44 (35, 56) | 0.597 |
| RASV, mL | 38 (31, 47) | 38 (31, 47) | 36 (28, 43) | **0.006** |
| RAEF, % | 47 (41, 53) | 47 (41, 53) | 44 (38, 50) | **0.002** |
| Max RAVi, mL/m^1.7^ | 45 (37, 53) | 45 (37, 53) | 42 (35, 51) | 0.058 |
| Min RAVi, mL/m^1.7^ | 23 (19, 29) | 23 (19, 29) | 23 (18, 29) | 0.834 |
| RASVi, mL/m^1.7^ | 21 (17, 25) | 21 (17, 25) | 19 (15, 23) | **<0.001** |
| GCS | -22 (-24, -20) | -22 (-24, -20) | -22 (-25, -20) | 0.609 |
| GRS | 45 (39, 50) | 45 (39, 50) | 44 (40, 51) | 0.775 |
| GLS | -18 (-20, -17) | -18 (-20, -17) | -18 (-20, -16) | 0.323 |
| GFI | 0.48 (0.43, 0.52) | 0.48 (0.43, 0.52) | 0.47 (0.41, 0.51) | **0.026** |
| **Medications any** (%) | 11290 (38.6) | 11142 (38.4) | 148 (73.6) | **<0.001** |
| Beta blockers (%) | 2068 (7.1) | 2011 (6.9%) | 57 (28.4) | **<0.001** |
| ACE (%) | 3316 (11.3) | 3251 (11.2%) | 65 (32.3) | **<0.001** |
| ARBi (%) | 1731 (5.9) | 1686 (5.8) | 45 (22.4) | **<0.001** |
| Loop diuretics/thiazides (%) | 1563 (5.3) | 1530 (5.3) | 33 (16.4) | **<0.001** |
| Calcium blockers (%) | 2990 (10.2) | 2929 (10.1) | 61 (30.3) | **<0.001** |
| MRA (%) | 91 (0.3) | 87 (0.3) | 4 (2.0) | **<0.001** |
| Nitrates (%) | 250 (0.9) | 238 (0.8) | 12 (6.0) | **<0.001** |
| Antilipidemic agents (%) | 6648 (22.7) | 6545 (22.5) | 103 (51.2) | **<0.001** |
| Antithrombotics (%) | 4525 (15.5) | 4452 (15.3) | 73 (36.3) | **<0.001** |
| Anticoagulants (%) | 273 (0.9) | 261 (0.9) | 12 (6.0) | **<0.001** |
| Antihyperglycemic agents (%) | 1004 (3.4) | 981 (3.4) | 23 (11.4) | **<0.001** |

Continuous variables were summarized as medians with interquartile ranges (IQRs). Categorical variables were reported as counts (n) and percentages (%). **CKD**: Chronic Kidney Disease; **BMI**: Body Mass Index; **IQR**: Interquartile Range; **LV**: Left Ventricle; **RV**: Right Ventricle; **LVEDV**: Left Ventricular End-Diastolic Volume; LVEDVi: left ventricular end-diastolic volume indexed to height^1.7^; **LVESV**: Left Ventricular End-Systolic Volume; **LVESVi**: Left Ventricular End-Systolic Volume indexed to height^1.7^; **LVEF**: Left Ventricular Ejection Fraction; **LVSV**: Left Ventricular Stroke Volume; **LVSVi**: Left Ventricular Stroke Volume indexed to height^1.7^; **LVCO**: Left Ventricular Cardiac Output; **LVCOi**: Left Ventricular Cardiac Output indexed to height^1.7^; **LVMi**: Left Ventricular Mass indexed to height^1.7^; **WT**: Wall Thickness; **WTi**: Wall Thickness indexed to height^1.7^**; RVSV**: Right Ventricular Stroke Volume; **RVSVi**: Right Ventricular Stroke Volume; **RVEF**: Right Ventricular Ejection Fraction; **RVEDV**: Right Ventricular End-Diastolic Volume; **RVEDVi**: Right Ventricular End-Diastolic Volume indexed to height^1.7^; **RVESV**: Right Ventricular End-Systolic Volume; **RVESVi**: Right Ventricular End-Systolic Volume indexed to height^1.7^; **Max LAV**: Left Atrial Volume (maximum); **Max LAVi**: Left Atrial Volume (maximum) indexed to height^1.7^; **LAEF**: Left Atrial Ejection Fraction; **LASV**: Left Atrial Stroke; Volume **LASV (i)**: Left Atrial Stroke Volume indexed to height^1.7^; **Max RAV**: Right Atrial Volume (maximum); **Max RAVi**: Right Atrial Volume (maximum) indexed to height^1.7^; **RAEF**: Right Atrial Ejection Fraction; **GCS**: Global Circumferential Strain; **GRS**: Global Radial Strain; **GLS**: Global Longitudinal Strain.

**Trend-level results are underlined. Statistically significant values are shown in bold**

# **Supplementary table 3: Baseline Characteristics of matched twins and their non-CKD controls**

| **Covariates** | **Twin Controls (N= 193)** | **Non-CKD Control (N=193)** | **P-values** | **SMD** |
| --- | --- | --- | --- | --- |
| Sex | 111 (57.5) | 111 (57.5) | 1.00 | <0.001 |
| Age | 68.00 (62.00, 72.00) | 68.00 (62.00, 73.00) | 0.969 | 0.002 |
| BMI, kg/m^2^ | 27.59 (24.71, 31.22) | 27.51 (24.57, 31.30) | 0.972 | 0.007 |
| Smoker, % | 4 (2.1%) | 4 (2.1%) | 1.00 | <0.001 |
| Ethnic Q-risk, % | 184 (95.3) | 184 (95.3) | 1.00 | <0.001 |
|  | 1 (0.5%) | 1 (0.5%) |  |  |
|  | 8 (4.1%) | 8 (4.1%) |  |  |
| Deprivation Townsend | -2.4 (-3.5, -0.9) | -2.4 (-3.60, -0.3) | 0.754 | 0.064 |
| Diabetes, % | 34 (17.6) | 34 (17.6) | 1.00 | <0.001 |
| Hyperlipidaemia, % | 111 (57.5) | 111 (57.5) | 1.00 | <0.001 |
| Hypertension, % | 136 (70.5) | 136 (70.5) | 1.00 | <0.001 |
| Any baseline Cardiac disease, % | 62 (32.1) | 62 (32.1) | 1.00 | <0.001 |
| Stroke, % | 13 (6.7) | 13 (6.7) | 1.00 | <0.001 |
| Global LV T1, ms | 919 (886, 954) | 919 (889, 952) | 0.541 | 0.089 |
| LVEDV, mL | 145 (119, 168) | 146 (129, 169) | 0.181 | 0.136 |
| LVEDVi, mL/m^1.7^ | 77 (66, 83) | 78 (69, 87) | 0.103 | 0.191 |
| LVESV, mL | 58 (45, 76) | 60 (50, 73) | 0.229 | 0.088 |
| LVESVi, mL/m^1.7^ | 30 (26, 37) | 32 (27, 37) | 0.119 | 0.123 |
| LVSV, mL | 86 (72, 98) | 86 (76, 98) | 0.264 | 0.156 |
| LVSVi, mL/m^1.7^ | 45 (40, 50) | 46 (41, 52) | 0.122 | 0.199 |
| LVEF, % | 59 (55, 63) | 58 (56, 63) | 0.744 | 0.014 |
| LVCO, L/min | 5.2 (4.3, 6.0) | 5.4 (4.8, 6.1) | **0.041** | 0.215 |
| LVCOi, L/min/m^1.7^ | 2.7 (2.4, 3.1) | 2.9 (2.5, 3.2) | **0.008** | 0.251 |
| LVM, g | 89 (71, 104) | 87 (75, 105) | 0.523 | 0.081 |
| WT, mm | 5.8 (5.3, 6.5) | 5.9 (5.4, 6) | 0.866 | 0.007 |
| LVMi, g/m^1.7^ | 46 (41, 52) | 47 (41, 53) | 0.31 | 0.126 |
| Max WT, mm | 7.2 (6.6, 8.2) | 7.2 (6.7, 8.1) | 0.922 | 0.005 |
| WTi, mm/m^1.7^ | 3.12 (2.84, 3.32) | 3.09 (2.92, 3.29) | 0.87 | 0.017 |
| Max WTi, mm/m^1.7^ | 3.86 (3.56, 4.12) | 3.81 (3.58, 4.18) | 0.984 | 0.014 |
| RVEDV, mL | 149 (127, 176) | 153 (136, 180) | 0.089 | 0.159 |
| RVEDVi, mL/m^1.7^ | 80 (69, 89) | 82 (74, 90) | **0.037** | 0.224 |
| RVESV, mL | 61 (50, 78) | 69 (56, 80) | 0.051 | 0.137 |
| RVESVi, mL/m^1.7^ | 33 (27, 41) | 35 (30, 41) | **0.035** | 0.193 |
| RVSV, mL | 84 (74, 99) | 87 (78, 101) | 0.144 | 0.137 |
| RVSVi, mL/m^1.7^ | 45 (40, 50) | 46 (42, 53) | 0.093 | 0.166 |
| RVEF, % | 58 (53, 62) | 57 (53, 60) | 0.178 | 0.108 |
| Max LAV, mL | 73 (55, 90) | 70 (58, 86) | 0.704 | 0.055 |
| Max LAVi, mL/m^1.7^ | 37 (30, 46) | 37 (30, 45) | 0.768 | 0.057 |
| Min LAVi, mL/m^1.7^ | 15 (11, 20) | 14 (11, 19) | 0.695 | 0.105 |
| Min LAV, mL | 28 (20, 38) | 27 (20, 37) | 0.64 | 0.101 |
| LASV, mL | 41 (33, 50) | 42 (36, 50) | 0.512 | 0.044 |
| LASVi, mL/m^1.7^ | 22 (18, 26) | 22 (19, 27) | 0.343 | 0.052 |
| LAEF, % | 60 (53, 65) | 61 (55, 66) | 0.403 | 0.127 |
| Max RAV, mL | 80 (65, 96) | 80 (69, 101) | 0.686 | 0.043 |
| Max RAVi, mL/m^1.7^ | 43 (34, 52) | 43 (36, 53) | 0.645 | 0.052 |
| RASV, mL | 36 (29, 46) | 38 (31, 46) | 0.171 | 0.14 |
| RASVi, mL/m^1.7^ | 19 (15, 23) | 20 (16, 24) | 0.122 | 0.13 |
| RAEF, % | 47 (39, 52) | 46 (42, 53) | 0.406 | 0.114 |
| Min RAV, mL | 42 (33, 56) | 42 (34, 56) | 0.85 | 0.023 |
| Min RAVi, mL/m^1.7^ | 22 (17, 29) | 22 (18, 29) | 0.874 | 0.013 |
| GCS | -22 (-25, -20) | -22 (-24, -19) | 0.332 | 0.085 |
| GRS | 45 (40, 51) | 45 (39, 49) | 0.592 | 0.043 |
| GLS | -18 (-20, -16) | -18 (-20, -16) | 0.716 | 0.047 |
| Medications, % | 132 (68) | 140 72.5) | 0.435 | 0.091 |
| Beta blockers, % | 43 (22.3) | 34 (17.6) | 0.308 | 0.117 |
| ACE, % | 54 (28.0) | 53 (27.5) | 1.00 | 0.012 |
| ARBi, % | 38 (19.7) | 33 (17.1) | 0.599 | 0.067 |
| Diuretics loop diuretics and thiazides, % | 28 (14.5) | 26 (13.5) | 0.883 | 0.03 |
| Calcium blockers | 48 (24.9) | 48 (24.9) | 1.00 | <0.001 |
| Nitrates, % | 8 (4.1) | 5 (2.6) | 0.573 | 0.086 |
| Antilipidemic agents, % | 99 (51.3) | 95 (49.2) | 0.76 | 0.041 |
| Antithrombotics, % | 61 (31.6) | 72 (37.3) | 0.284 | 0.12 |
| Anti-coagulants, % | 9 (4.7) | 2 (1.0) | 0.066 | 0.219 |
| Anti-hyperglycaemic agents, % | 13 (6.7) | 18 (9.3) | 0.454 | 0.095 |

Continuous variables were summarized as medians with interquartile ranges (IQRs). Categorical variables were reported as counts (n) and percentages (%). **CKD**: Chronic Kidney Disease; **BMI**: Body Mass Index; **IQR**: Interquartile Range; **LV**: Left Ventricle; **RV**: Right Ventricle; **LVEDV**: Left Ventricular End-Diastolic Volume; LVEDVi: left ventricular end-diastolic volume indexed to height^1.7^; **LVESV**: Left Ventricular End-Systolic Volume; **LVESVi**: Left Ventricular End-Systolic Volume indexed to height^1.7^; **LVEF**: Left Ventricular Ejection Fraction; **LVSV**: Left Ventricular Stroke Volume; **LVSVi**: Left Ventricular Stroke Volume indexed to height^1.7^; **LVCO**: Left Ventricular Cardiac Output; **LVCOi**: Left Ventricular Cardiac Output indexed to height^1.7^; **LVMi**: Left Ventricular Mass indexed to height^1.7^; **WT**: Wall Thickness; **WTi**: Wall Thickness indexed to height^1.7^**; RVSV**: Right Ventricular Stroke Volume; **RVSVi**: Right Ventricular Stroke Volume; **RVEF**: Right Ventricular Ejection Fraction; **RVEDV**: Right Ventricular End-Diastolic Volume; **RVEDVi**: Right Ventricular End-Diastolic Volume indexed to height^1.7^; **RVESV**: Right Ventricular End-Systolic Volume; **RVESVi**: Right Ventricular End-Systolic Volume indexed to height^1.7^; **Max LAV**: Left Atrial Volume (maximum); **Max LAVi**: Left Atrial Volume (maximum) indexed to height^1.7^; **LAEF**: Left Atrial Ejection Fraction; **LASV**: Left Atrial Stroke; Volume **LASV (i)**: Left Atrial Stroke Volume indexed to height^1.7^; **Max RAV**: Right Atrial Volume (maximum); **Max RAVi**: Right Atrial Volume (maximum) indexed to height^1.7^; **RAEF**: Right Atrial Ejection Fraction; **GCS**: Global Circumferential Strain; **GRS**: Global Radial Strain; **GLS**: Global Longitudinal Strain.

**Trend-level results are underlined. Statistically significant values are shown in bold**

# **Supplementary table 4. Reverse-Pair Analysis: Myocardial T1 in Non-CKD Controls with Adverse Outcomes**

| Outcome | N (pairs) | Mean Difference (ms) | 95% CI (ms) | SD | P-value |
| --- | --- | --- | --- | --- | --- |
| All-cause mortality | 3 | -49 | -382, 283 | 134 | 0.591 |
| Cardiovascular mortality | 2 | -77 | -1662, 1507 | 176 | 0.645 |
| Myocardial infarction | 7 | -2 | -73, 68 | 76 | 0.938 |
| Heart failure | 4 | -9 | -51, 32 | 26 | 0.533 |
| Atrial fibrillation | 6 | -21 | -97, 54 | 72 | 0.505 |

# **Supplementary Table 5. Associations Between Myocardial Native T1, CKD Status, and Incident Outcomes Using Stratified Cox Regression Models**

| **Outcome** | **Predictor** | **HR (95% CI)** | **p-value** | **Model p-value** |
| --- | --- | --- | --- | --- |
| All-cause mortality | T1_scaled | 2.02 (0.46, 8.82) | 0.35 | 0.02 |
| All-cause mortality | CKD status | 8.06 (0.84, 77.02) | 0.07 |  |
| All-cause mortality | T1_scaled × CKD status | 2.02 (0.19, 21.74) | 0.56 |  |
| CV death | T1_scaled | 1.25e5 (0,inf) | 0.999 | 0.2 |
| CV death | CKD status | 1.05e3 (0, Inf) | 1 |  |
| CV death | T1_scaled × CKD status | 5.05e-5 (0, Inf) | 0.999 |  |
| Myocardial Infarction | T1_scaled | 3.25 (0.13, 82.10) | 0.48 | 0.5 |
| Myocardial Infarction | CKD status | 0.17 (0.01, 4.65) | 0.29 |  |
| Myocardial Infarction | T1_scaled × CKD status | 0.07 (0.00009, 53.70) | 0.43 |  |
| Atrial Fibrillation | T1_scaled | 3.79 (0.61, 23.68) | 0.15 | 0.4 |
| Atrial Fibrillation | CKD status | 2.13 (0.19, 24.44) | 0.55 |  |
| Atrial Fibrillation | T1_scaled × CKD status | 0.23 (0.01, 4.97) | 0.35 |  |
| Heart Failure | T1_scaled | 1.69 (0.31, 9.13) | 0.54 | 0.06 |
| Heart Failure | CKD status | 2.42 (0.39, 15.14) | 0.34 |  |
| Heart Failure | T1_scaled × CKD status | 2.28 (0.18, 29.07) | 0.52 |  |

Models evaluate the association between myocardial native T1 (scaled per 1 SD increase), CKD status, and their interaction (**T1 × CKD**) with incident outcome

# **Supplementary Table 6. Associations of Myocardial Native T1 with Incident Outcomes in CKD Cases and Matched Non-CKD Controls: Results from Penalised Cox Regression**

| **Outcome** | **Events** | **HR 95% CI** | **P-value** |
| --- | --- | --- | --- |
| Atrial Fibrillation | 6 | 2.08 (1.01, 4.04) | **0.048** |
| Myocardial Infarction | 5 | 0.73 (0.33, 1.75) | 0.51 |
| Cardiovascular Death | 3 | 3.67 (1.69, 8.95) | **0.002** |

#

# **Supplementary figures**

# **Supplementary Figure 1. The Covariate Balance plot: Comparison of the balance of covariates between CKD cases and control groups before and after matching.**


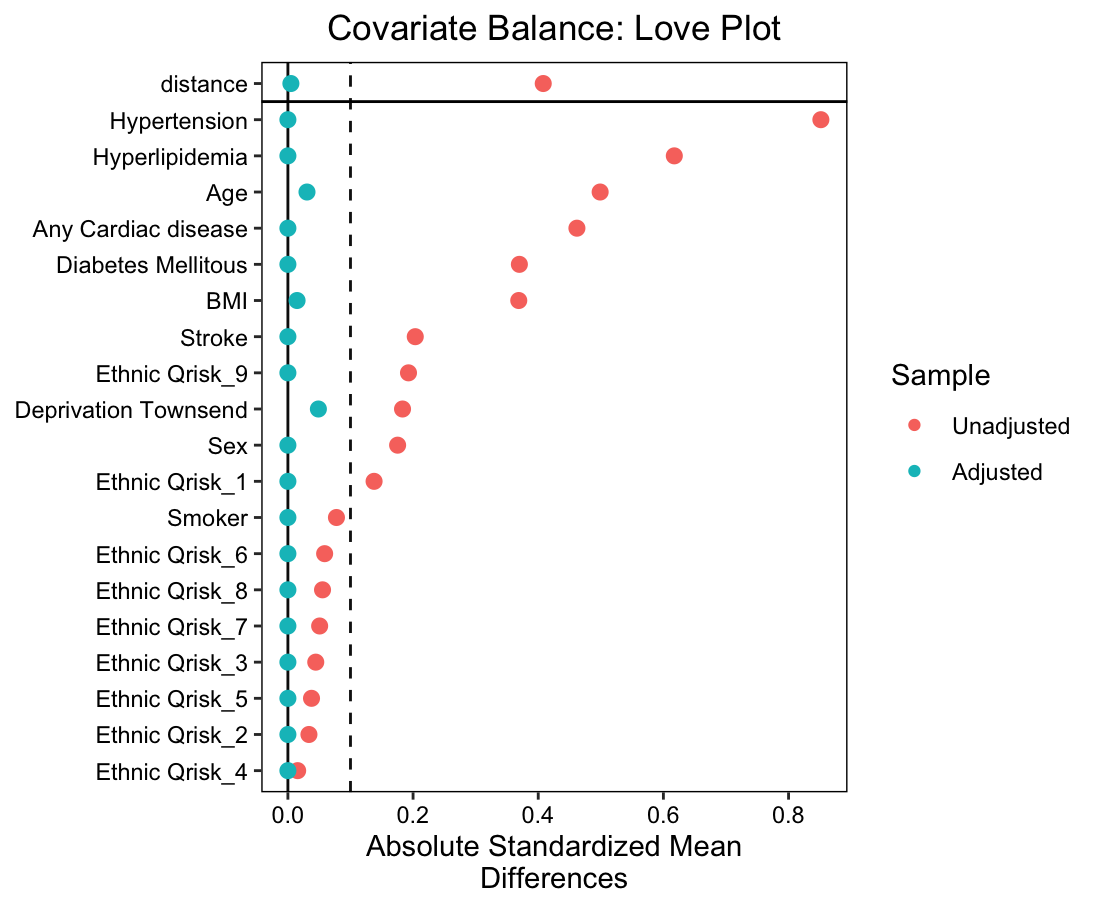


**Red Dots (Unadjusted):**

Represent the absolute mean differences of covariates before matching between treated and control groups.

**Green Dots (Adjusted):** Represent the absolute mean differences of covariates after matching between treated and control groups.

**X-Axis - Absolute Mean Differences:**

The x-axis shows the absolute mean differences between groups. The values represent the standardized mean differences (SMDs) for each covariate. Smaller values indicate better balance, meaning that the covariate distributions between the CKD and non-CKD groups are similar.

**Dashed Line at 0.1 (Threshold):**

The dashed vertical line at 0.1 indicates the generally accepted threshold for good balance.

SMD < 0.1 suggests that the covariate is well-balanced between the treatment and control groups. Ideally, the green dots (after matching) should be as close to or to the left of this threshold line as possible.

**Covariate Names (y-axis):**

The covariates listed on the y-axis are those that were included in the matching model.

Each covariate has an associated unadjusted (red) and adjusted (green) dot representing its balance before and after matching.
